# Design of Safe and Efficient Adenine Base Editors via Protein Language Model Screening for Osteoarthritis Treatment

**DOI:** 10.1002/advs.202519807

**Published:** 2026-04-29

**Authors:** Jiawei Yao, Dalin Chen, Ziyi Zhang, Jingxuan Ren, Chengcheng Zhao, Shengfang Wang, Mengyu Shang, Dawei Jiang, Yinuo Li, Su'an Tang, Kai Li, Xiaohui Zhang, Xiaogang Wang

**Affiliations:** ^1^ Guangdong Provincial Key Laboratory of Bone and Joint Degenerative Diseases The Third Affiliated Hospital Southern Medical University Guangzhou China; ^2^ State Key Laboratory of Common Mechanism Research for Major Diseases Suzhou Institute of Systems Medicine Chinese Academy of Medical Sciences & Peking Union Medical College Suzhou China; ^3^ Department of Spinal Surgery, Orthopedic Medical Center Zhujiang Hospital, Southern Medical University Guangzhou China

**Keywords:** base editor, minimal off‐target, protein language model, rational design

## Abstract

Base editors enable precise genome modification and have emerged as a promising therapeutic approach for correcting diseases caused by single‐nucleotide variants. While the current efficient version of adenine base editors (ABEs), such as ABE8e, exhibits exceptional efficiency for A‐to‐G conversions, their clinical translation is hindered by persistent high off‐target editing effects. Here, we applied artificial intelligence‐assisted design a safe ABE variant, RDLot‐ABE, with a narrow(4 nt) editing window and substantially lower DNA off‐target editing activity compared to ABE8e. Moreover, targeted knockdown of *Fscn1* for osteoarthritis treatment using RDLot‐ABE alleviates cartilage degradation in explants derived from human patients. Notably, intra‐articular delivery of the RDLot‐ABE to reduce *Fscn1* effectively arrests disease progression in a murine osteoarthritis model. These findings establish RDLot‐ABE as a safe and precise tool, expanding the clinical potential of gene editing therapies.

## Graphical abstract

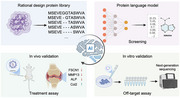

## 1 Introduction

Single‐nucleotide variants (SNVs) are responsible for approximately 58% of known human genetic diseases, with nearly half of these cases (47%) attributed to C•G‐to‐T•A transitions [1]. Correcting these mutations can prevent and even treat related diseases. CRISPR‐guided base editors are engineered by fusing a deaminase domain with a catalytically impaired Cas9, offering potent single‐nucleotide modification capabilities on DNA. Crucially, they achieve this without inducing double‐strand breaks (DSBs), thereby minimizing the risks of large‐scale chromosomal rearrangements and indel formation [2, 3, 4]. Consequently, they have emerged as powerful tools for correcting pathogenic mutations. So far, two main categories of base editors have been established: Adenine Base Editors (ABEs) for A•T to G•C transitions and Cytidine Base Editors (CBEs) for C•G to T•A transitions [5].

Although the repertoire of base editors has expanded with improved efficiency and broader targeting ranges, their enhanced deaminase activity has introduced risks of off‐target editing [6]. Even so, given their more safety editing outcomes compared to CBEs, ABEs remain the preferred class of editors in mammalian studies, driving the development of potent variants to ensure in vivo efficiency [4, 7]. For instance, the hyperactive ABE8e tends to induce unintended mutations during target editing due to its excessively broad editing activity. This off‐target activity remains a critical risk for its therapeutic application. Therefore, developing ABE variants that minimize off‐target activity is essential to address this challenge.

Recently, researchers have made substantial progress in optimizing ABEs to address this challenge, including rational engineering [8, 9, 10, 11], incorporation of DNA‐binding domain [12], directed evolution [13, 14], and gRNA engineering [15, 16]. However, the construction of large‐scale libraries and the subsequent screening of extensive base editor variants remain notoriously labor‐intensive and time‐consuming [17]. Consequently, developing a computational design strategy is essential to accelerate the engineering of base editors with reduced off‐target activity. Although Protein Language Models (PLMs) have shown remarkable success in capturing evolutionary fitness, purely data‐driven optimization is often agnostic to specific functional requirements like specificity [18]. To this end, integrating the rational design with the PLMs presents a compelling strategy.

**FIGURE 1:**
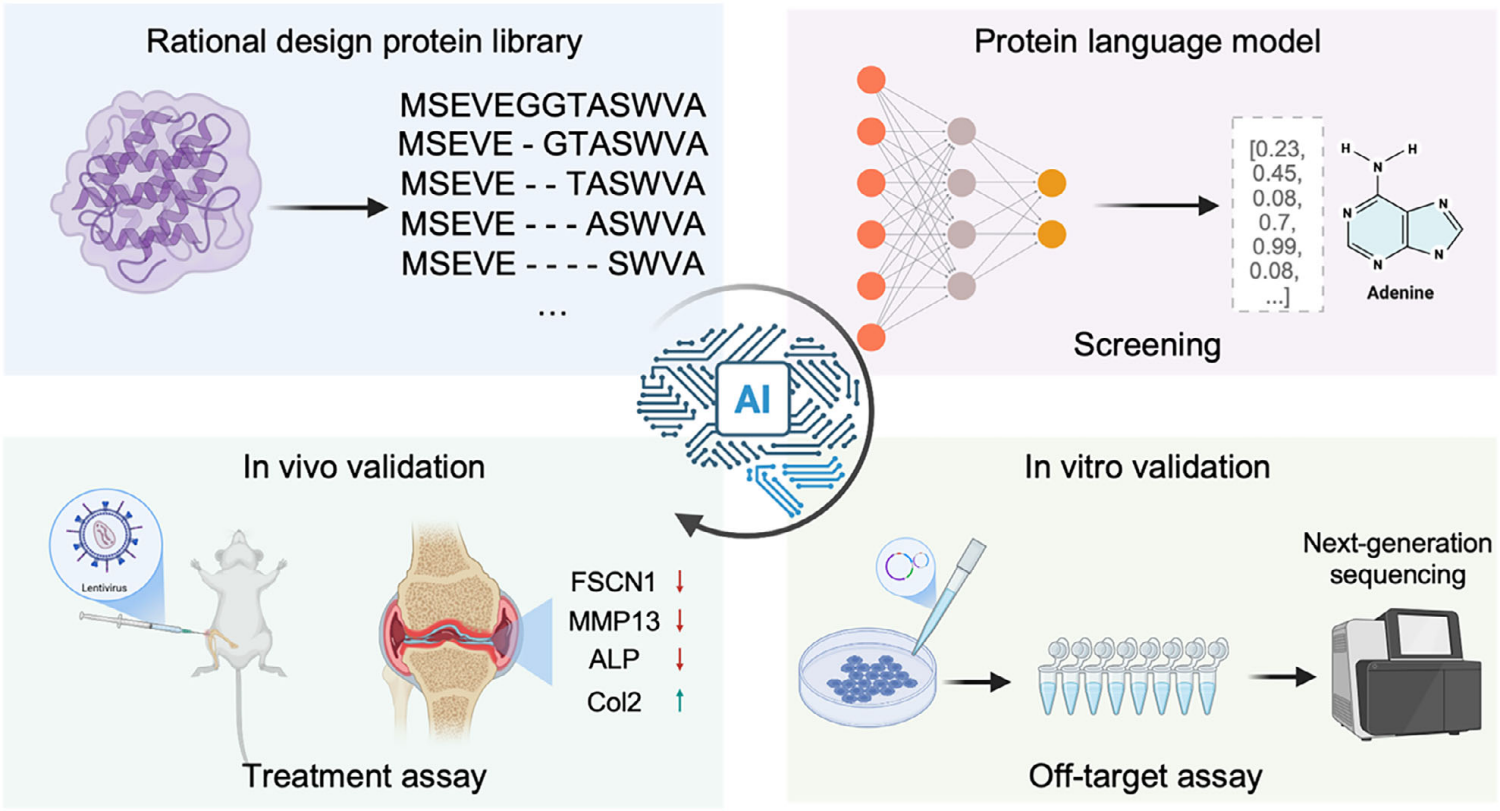
Workflow of the RDLot‐ABE design process. By shortening the amino acid sequences between interactive regions and the size of the editing enzyme was minimized. Subsequently, an artificial intelligence‐based structural embedding large language model was constructed to screen for potential high‐specificity editing enzyme variants. High‐throughput sequencing was used to verify the features of RDLot‐ABE variants in gene editing, and A‐to‐G base editing was effectively achieved at the GT splice donor site of the *Fscn1* gene, reducing *Fscn1* expression and OA phenotypes, demonstrating potential clinical application value.

In this study, we employed a screening strategy that integrates structure‐based rational design with PLMs. Specifically, we constructed a library of internal deletion variants designed to sterically constrict the active site, and prioritized candidates using a multimodal deep learning framework that synergizes evolutionary sequence embeddings with geometric structural features. Utilizing this strategy, we screened and identified a lead candidate, RDLot‐ABE (Rationally Designed Low‐off‐target ABE), featuring a deletion of residues 70–74. This variant exhibits a refined 4‐nt editing window and off‐target activity substantially lower than that of the ABE8e, while maintaining editing efficiency with no significant difference compared to the ABE8e. To evaluate therapeutic potential, we targeted *Fscn1*, a previously identified driver gene of Osteoarthritis (OA) that is specifically upregulated in the superficial zone of cartilage and promotes cartilage degeneration [19]. We show that RDLot‐ABE effectively disrupting *Fscn1* expression and mitigating OA phenotypes in both mice models and human‐derived cartilage explants (Figure 1). Overall, our work establishes an ABE variant that retains sufficient therapeutic potency to execute robust disease correction with a safety profile in models. These findings further emphasize the huge potential of AI‐assisted base editor precision design in a wide range of gene therapy applications.

## 2 Result

### 2.1 Rational Construction of TadA‐8e Variants Library

Conventional strategies to optimize features have largely focused on introducing amino acid mutations to reinforce protein–DNA interactions in regions proximal to the catalytic site [9, 11]. In our previous work, we have demonstrated that partial truncations of TadA‐8e can effectively modulate enzymatic precision by exposing latent DNA‐binding interfaces and inducing favorable conformational rearrangements [20]. However, while such miniaturized variants are highly advantageous for size‐constrained delivery systems, deletions impinging upon core binding motifs frequently compromise enzymatic function, underscoring the structural sensitivity of these regions. Leveraging these insights, we sought to refine enzyme fidelity through structural perturbations via partial deletions while preserving the core DNA‐binding interface of TadA‐8e.

**FIGURE 2:**
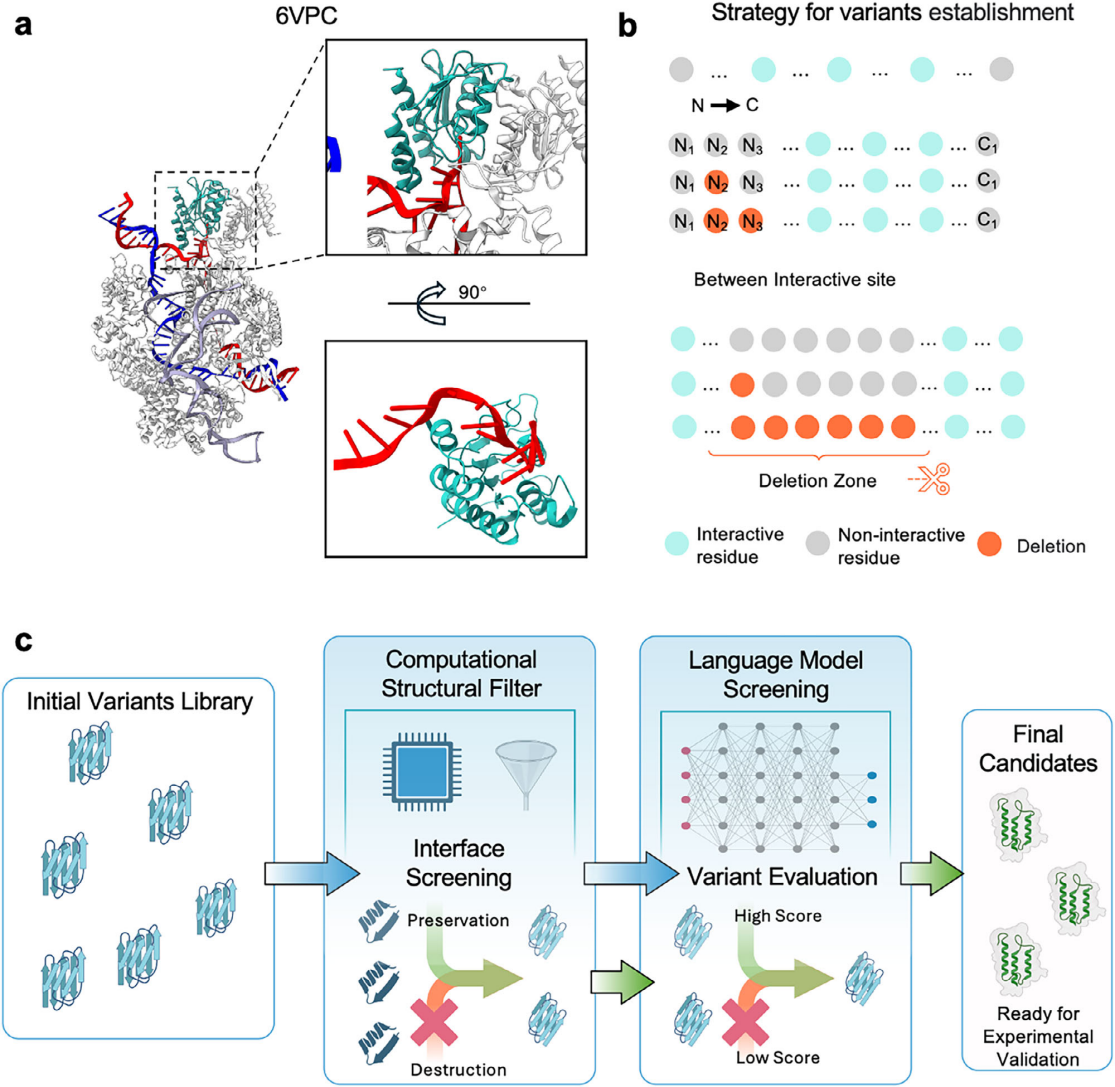
Structure‐guided design and computational screening pipeline of the truncated TadA‐8e library. (a) Structural basis for rational library design. Schematic representation of the molecular interplays between TadA‐8e (cyan) and the single‐stranded DNA substrate (red sticks), based on the cryo‐EM structure (PDB: 6VPC). Key DNA‐binding interfaces were identified to delineate regions essential for catalytic activity. (b) Construction of the virtual truncation library. Schematic of the strategy used to generate 1674 variants. Systematic amino acid deletions were introduced at the N‐terminus, C‐terminus, and the intervening loop segments between core DNA‐interaction domains to construct the virtual truncation library. (c) Computational screening and prioritization pipeline. The integrated workflow for identifying high‐performance variants: (i) structural‐guided rational construction of the virtual library; (ii) high‐throughput structural filtration using ESMFold to eliminate misfolded or unstable candidates; and (iii) functional scoring and ranking via the specialized protein language model to prioritize variants for downstream experimental validation.

To minimize off‐target effects through active‐site spatial constriction, we engineered a library of 1674 variants via targeted sequence shortening. Guided by the TadA‐8e/DNA complex structure (PDB: 6VPC) [21], we strategically preserved critical DNA‐interaction domains (residues 45‐61, 83–91, 104–118, and 149–157) while introducing deletions within the intervening loops connecting secondary structures (Figure 2a). By coupling well‐tolerated N‐ and C‐terminal truncations with internal deletions designed to induce functional conformational perturbations (Figure 2b), we generated a sequence library. To navigate this library and prioritize candidates with optimal structural integrity and catalytic retention, we developed a specialized computational screening pipeline (Figure 2c).

### 2.2 Structure‐Aware Protein Language Model‐Guided Virtual Screening of RDLoT‐ABE Variants

**FIGURE 3:**
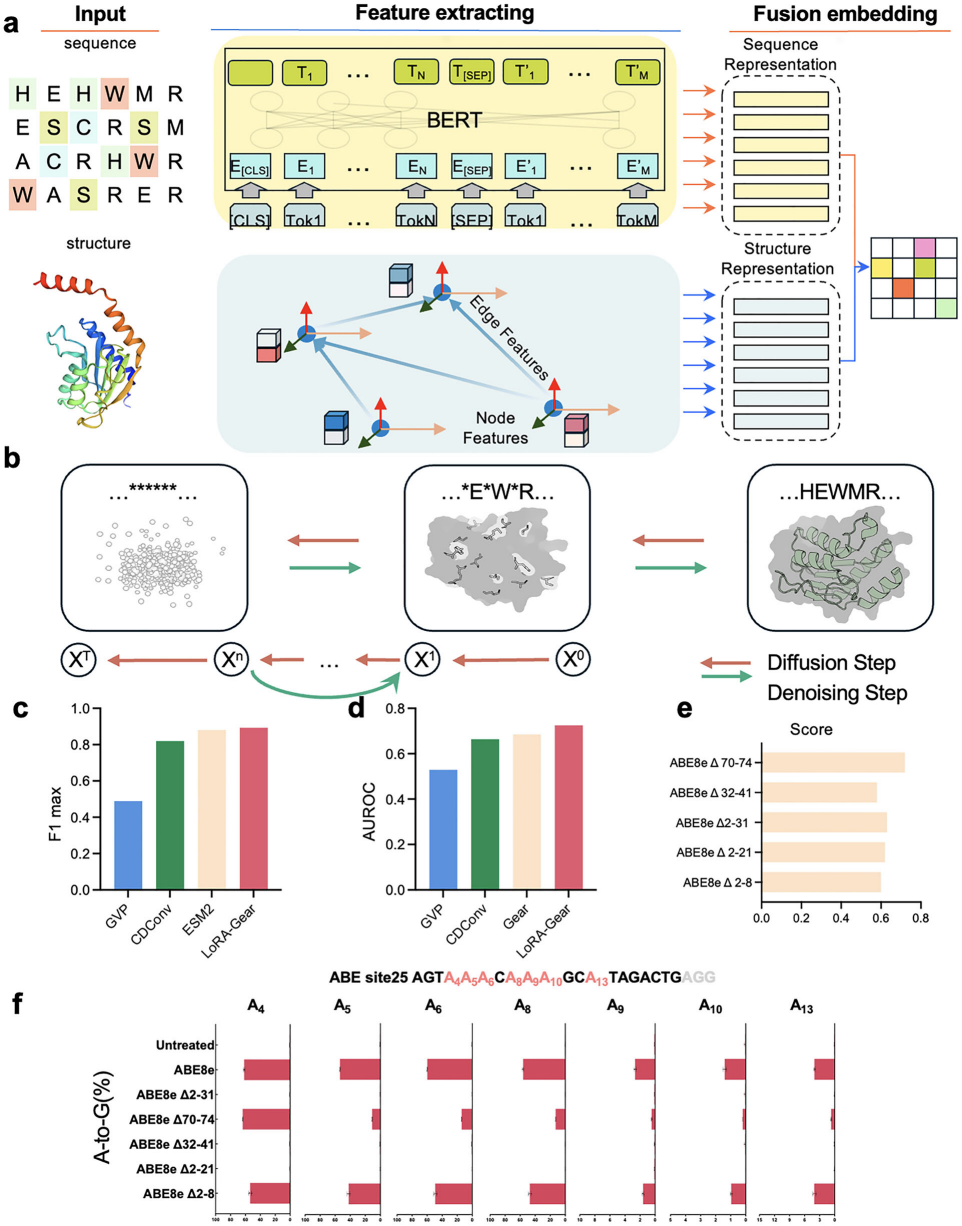
Construction and validation of the LoRA‐Gear framework and primary screening of high‐fidelity variants. (a) Schematic of the LoRA‐Gear architecture. The model employs a multimodal input strategy, utilizing a BERT‐like PLM to process sequence information and a Graph Convolutional Network (GCN) to extract geometric features from 3D structural coordinates. A fusion module integrates evolutionary semantic embeddings with spatial geometric features to achieve a comprehensive representation of protein function. (b) Denoising pre‐training mechanism based on diffusion models. The pre‐training workflow on the AFDB dataset is illustrated: protein sequences and structures are initially subjected to random masking, followed by iterative denoising steps to progressively recover original sequence features and atomic coordinates, thereby enhancing the model's performance of the protein representation. (c) The performance of the model in the EC classification task. In a benchmark encompassing 482 enzyme categories, LoRA‐Gear consistently outperformed baseline models, including CDConv, GVP, and ESM‐2, across both F1 max score. (d) The performance of the model in the MSP prediction task. The model's capacity to capture the impact of mutations on protein stability was evaluated using AUROC, confirming its robustness for downstream functional fine‐tuning. (e) Scoring of the virtual library. All candidates were ranked using the fine‐tuned LoRA‐Gear model to identify variants with high potential for enhanced editing specificity. (f) The efficiency of A‐to‐G the top 5 variants were examined at an endogenous genomic site (ABE site25) containing multiple adenosines within the editing window in HEK293T cells, with ABE8e serving as controls. Data are mean ± s.d. (*n* = 3 independent experiments).

To navigate the variant library, we developed LoRA‐Gear, a multimodal deep learning framework that integrates ESM2 embeddings with structure‐based geometric features (Figure 3a). By incorporating a Low‐Rank Adaptation (LoRA) [22] module and leveraging diffusion‐based pre‐training [23, 24] (Figure 3b), LoRA‐Gear outperformed established models (CDConv [25], GVP [26], and GearNet [27]) in predicting enzyme functions [28] and mutational stability [27] (Figure 3c,d). These findings attest to the model's advanced ability to capture protein features, indicating its potential to be effectively fine‐tuned for a broader spectrum of downstream tasks. Accordingly, we fine‐tuned LoRA‐Gear on an ABE‐specific dataset to capture the fitness landscape of editing specificity, enabling the precise identification of high‐fidelity variants. We then implemented a two‐stage screening pipeline: this involved an initial structural filtration via ESMFold to filter out structure with plDDT < 80, followed by LoRA‐Gear‐based ranking to prioritize candidates for experimental validation.

Following the computational screening pipeline, we selected the top five candidates for experimental validation at an endogenous target (ABE site 25) harboring multiple adenines. After co‐transfecting ABE mutants and ABE site25 sgRNA in HEK293T cells for 72 h, the genomic DNA from the cells for amplicon high‐throughput sequencing (HTS).  The results revealed that one specific ABE8e variant, ABE8e Δ70‐74(RDLot‐ABE), exhibited adenine editing potency comparable to that of wild‐type ABE8e at the A_4_ position of this locus. Compared to the extensive A_6_–A_9_ window of ABE8e and ABE8e Δ2‐8, RDLot‐ABE demonstrates enhanced precision, characterized by minimized bystander effects and a refined major editing window centered at A_4_(Figure 3e,f). To elucidate the structural basis of this performance, AlphaFold3 [29] prediction revealed that RDLot‐ABE possesses a narrowed active‐site geometry (Figure S3a,b), characterized by enhanced global structural stability and a constricted catalytic pocket (Figure S3a,c,d). We hypothesize that this steric constraint reduces non‐specific DNA engagement, thereby improving fidelity. Consequently, we selected RDLot‐ABE for further experimental validation.

### 2.3 Characterization of RDLot‐ABE in Mammalian Cells

**FIGURE 4:**
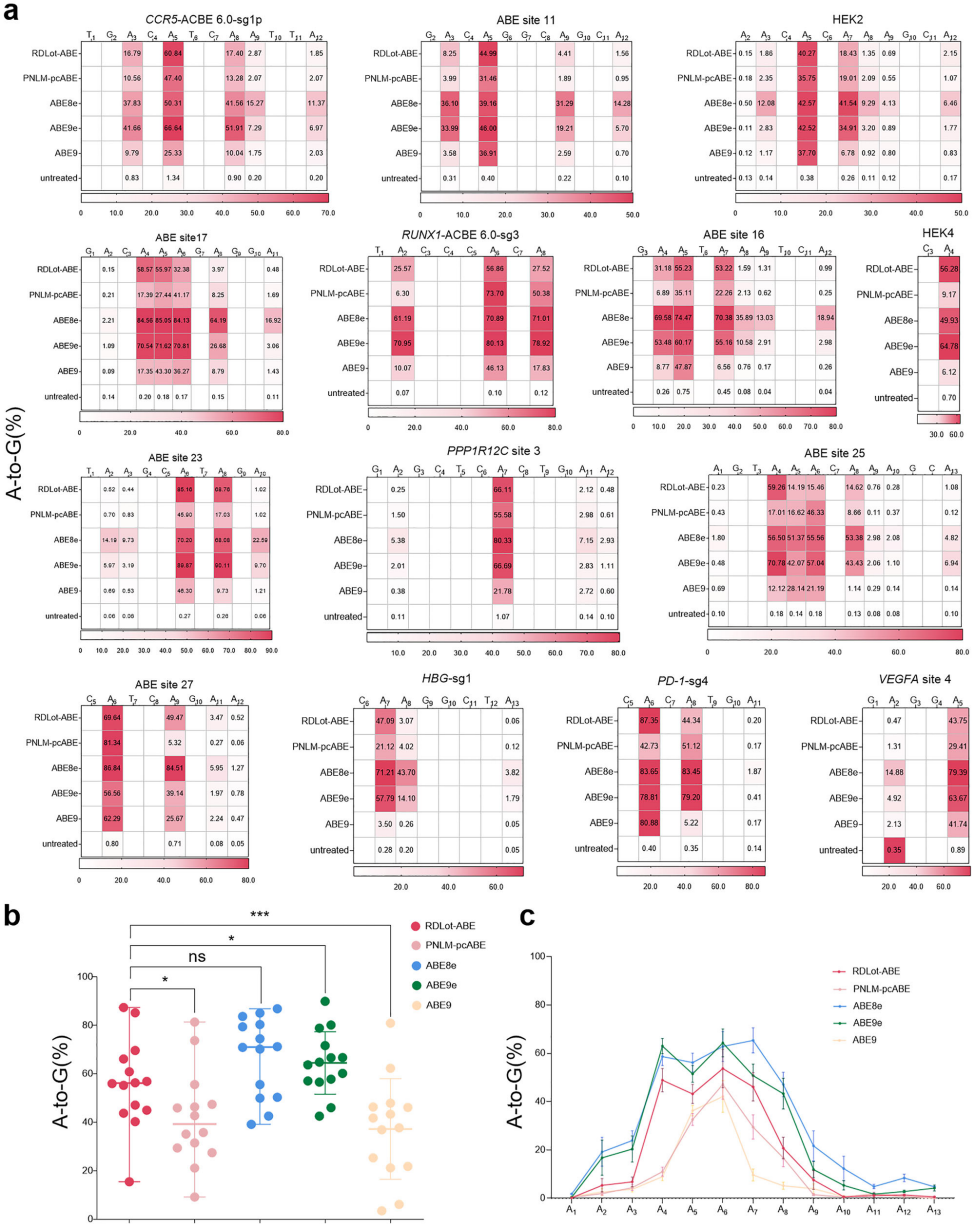
The characterization of RDLot‐ABE. (a) The A‐to‐G base editing efficiency of ABE8e, ABE9, ABE9e, PNLM‐pcABE, and RDLot‐ABE were examined at 14 endogenous genomic loci containing multiple As in HEK293T cells. The heatmap reflects averaged data from three biological replicates. (b) The most efficiently edited adenine within the activity window was analyzed across 14 endogenous genomic loci. Data are presented as mean ± s.d. (*n* = 3 independent experiments). *Pp*‐values were determined by a two‐sided paired Wilcoxon rank‐sum test istical significance was defined as ns, not significant; *p < 0.05; **p < 0.01; ***p < 0.001. (c) The average A‐to‐G base editing efficiency of ABE8e, ABE9, ABE9e, PNLM‐pcABE, and RDLot‐ABE at 14 endogenous genomic loci containing multiple As in Figure 4a. Data are mean ± s.d. (*n* = 3 independent experiments).

To further characterize RDLot‐ABE, we tested 14 endogenous target sites in HEK293T cells with ABE8e, ABE9e [30], ABE9, and PNLM‐pcABE as controls. The results showed all base editors enable efficient A‐to‐G base editing with RDLot‐ABE from 15.46% to 87.35%, ABE8e from 39.16% to 83.65%, ABE9e from 38.52% to 89.87%, PNLM‐pcABE from 9.17% to 73.70%, and ABE9 from 3.50% to 80.88% by analyzing the highest‐editing‐efficiency adenine sites across all 14 targets. Notably, RDLot‐ABE exhibits comparable editing efficiency to ABE8e and ABE9e, while achieving significantly higher base editing efficiency than both ABE9 and PNLM‐pcABE (Figure 4a,b). The editing window of RDLot‐ABE was A_4_–A_7_, which was narrower than those of ABE8e (A_2_–A_9_) and ABE9e (A_2_–A_8_), and slightly wider than those of ABE9 (A_5_–A_6_) and PNLM‐pcABE (A_5_–A_7_) (Figure 4c). Therefore, RDLot‐ABE was also a precise and highly efficient adenine base editor.

### 2.4 Off‐Target Assessment of RDLot‐ABE in Mammalian Cells

**FIGURE 5:**
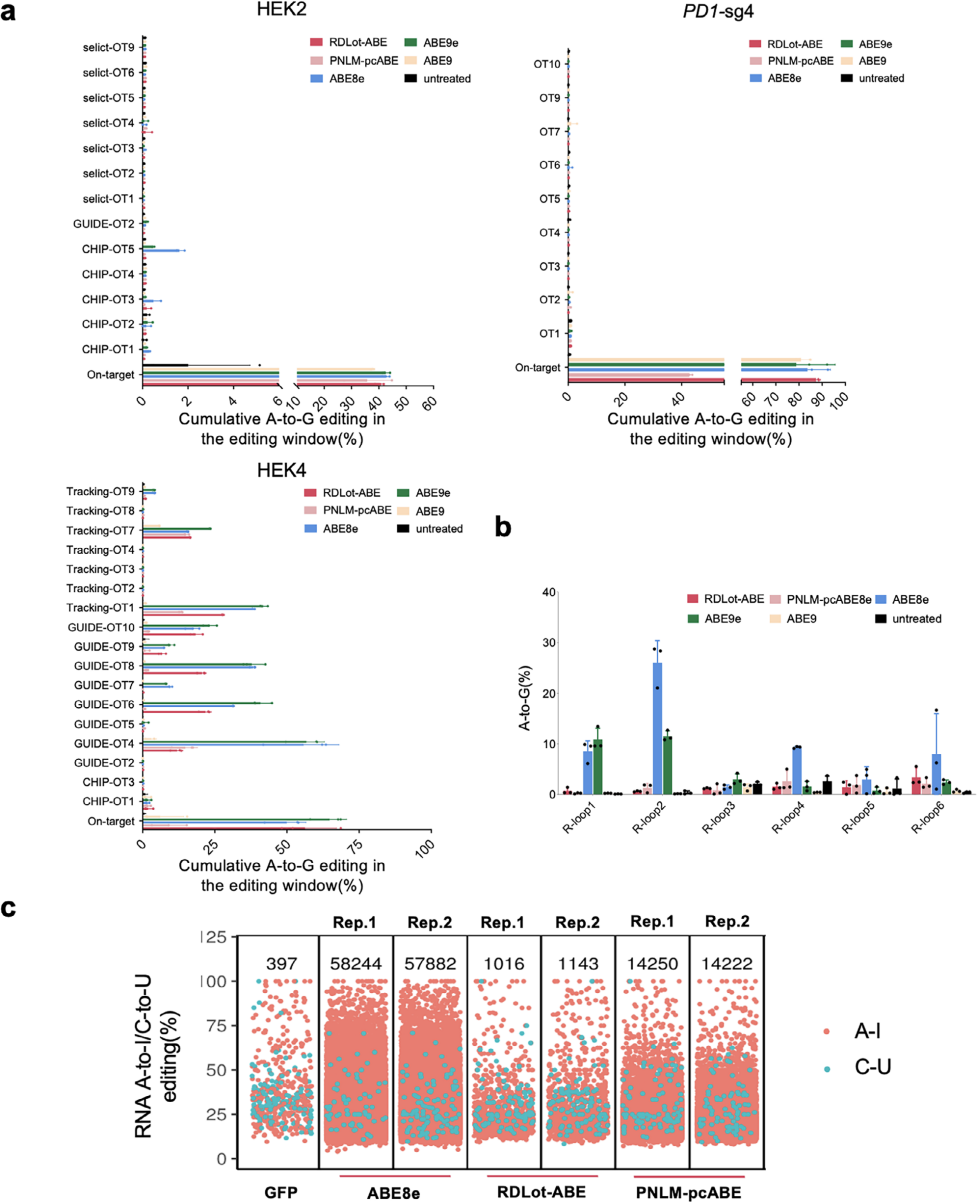
Off‐target assessment of RDLot‐ABE. (a) Cas9‐dependent DNA off‐target analysis at the indicated targets (HEK site2, PD‐1‐sg4, and HEK site4) of ABE8e, ABE9, ABE9e, PNLM‐pcABE, and RDLot‐ABE in HEK293T cells. Data are means ± s.d. (*n*  =  3 independent experiments). (b) Cas9‐independent DNA off‐target analysis of the modified orthogonal R‐loop by ABE8e, ABE9, ABE9e, PNLM‐pcABE, and RDLot‐ABE. Data are means ± s.d. (*n*  =  3 independent experiments). (c) RNA off‐target editing activity by ABE8e, PNLM‐pcABE, and RDLot‐ABE using RNA‐seq, GFP is negative control. The red and green data points in the figure represent A‐to‐I and C‐to‐U, respectively. Each biological replicate is listed on the top.

We subsequently assessed off‐target effects of RDLot‐ABE through three approaches: sgRNA‐dependent DNA off‐target, sgRNA‐independent DNA off‐target, and RNA off‐target. First, for sgRNA‐dependent DNA off‐target evaluation, 39 off‐targets in total were selected‐9 of which were in silico predicted off‐target sites from *PD‐1*‐sg4 using Cas‐OFFinder [31], 16 were from previously known Cas9 off‐target sites (HEK site2 and HEK site4) identified by GUIDE‐seq [32] or ChIP‐seq [33], and 14 were from previously known ABE off‐target sites (HEK site2 and HEK site4) identified by Selict‐seq [34] or Tracking‐seq [35]. The results showed that 10 out of 39 exhibited off‐target editing events for ABE8e and ABE9e, and only 3 out of 39 exhibited off‐target editing events for PNLM‐pcABE, 2 out of 39 exhibited off‐target editing events for ABE9, and 7 out of 39 exhibited off‐target editing events for RDLot‐ABE, respectively (Figure 5a). At these off‐target sites, RDLot‐ABE exhibits reduced off‐target editing efficiency relative to ABE8e and ABE9e, while showing comparable off‐target editing efficiency to ABE9 and PNLM‐pcABE (Figure 5a). Second, for sgRNA‐independent DNA off‐target, the Modified R‐loop assay [36] was used for evaluation. The results showed RDLot‐ABE, similar to ABE9 and PNLM‐pcABE, have no off‐target editing events, with a superior performance than that of ABE8e and ABE9e (Figure 5b). Third, for RNA off‐target, the whole‐transcriptomic RNA sequencing was evaluated. The results showed RDLot‐ABE exhibited near background RNA off‐target events, which has far lower off‐target events than that of ABE8e and PNLM‐pcABE(Figure 5c). In summary, RDLot‐ABE represents a base editing tool that combines high efficiency with extremely low off‐target effects, making it a promising platform for future therapeutic applications.

### 2.5 RDLot‐ABE Mediates Silencing of the OA Driver Gene *Fscn1* in Vitro

OA is the most common disabling arthropathy that affects the whole joint and leads to chronic disability and functional impairment. We have previously reported that FSCN1 is significantly increased in the superficial zone of damaged OA articular cartilage and that targeted silencing of this gene attenuates disease progression [19]. Designing a strategy to downregulate *Fscn1* by targeting its splice donor sequence, providing a potential gene therapy intervention for OA.

**FIGURE 6:**
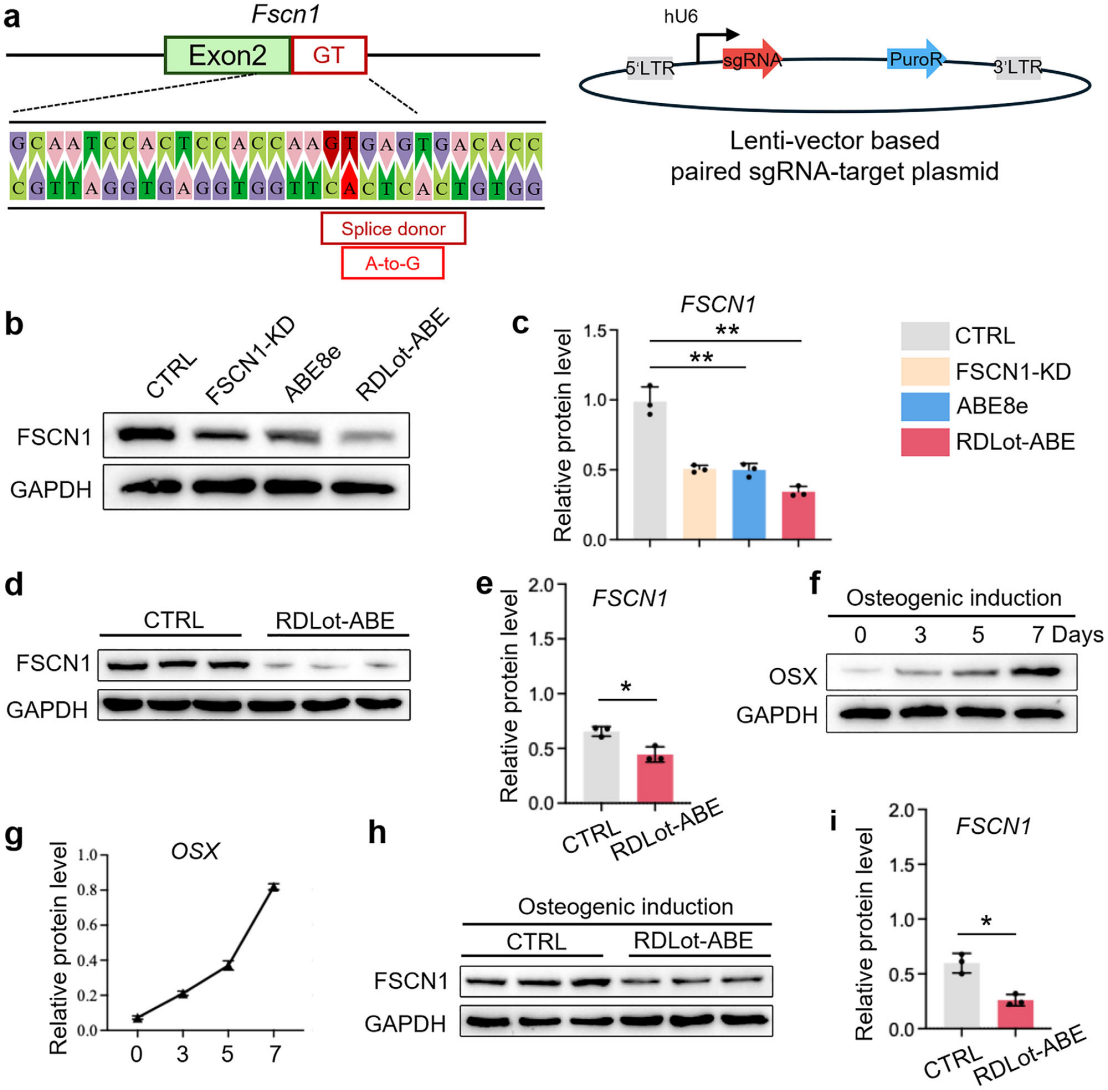
The effectiveness of RDLot‐ABE adenoviral therapy in silencing *Fscn1* in chondrocytes, synovial fibroblasts, and osteoblasts. (a) The splice donor sequence of exon2 of the mouse *Fcsn1* gene targeted by RDLot‐ABE. The splice donor site “GT” is shown in red(left). Schematic diagram of lenti‐vector‐based paired sgRNA‐target plasmid(right). (b,c) Western blot and quantitative analysis of FSCN1 protein levels in mice chondrocytes treated with CTRL, FSCN1‐targeting shRNA (FSCN1‐KD), ABE8e, or RDLot‐ABE. (d,e) Western blot and quantification analysis of FSCN1 protein levels in mice synovial fibroblasts treated with CTRL or RDLot‐ABE. (f,g) Time‐course Western blot analysis of OSX protein expression in mice BMSCs at days 0, 3, 5, and 7 during osteogenic induction. (h,i) Western blot and quantification of FSCN1 protein levels in osteoblasts treated with CTRL or RDLot‐ABE at day 7. Experiments were repeated three (c – i) times with similar results. Quantitative data are shown as means ± s.d. Significance level are indicated as follows: *p < 0.05, and **p < 0.01.

To evaluate the effect of our targeting strategy in vitro, we employed the RDLot‐ABE system to disrupt the conserved GT splice donor site within exon 2 of *Fscn1* across several multiple OA‐relevant murine joint cell types (Figure 6a). Western blot analysis at 72 h post‐transfection revealed that RDLot‐ABE markedly reduced FSCN1 protein levels. Notably, this silencing effect was superior to that of both the ABE8e editor and shRNA‐mediated knockdown controls (Figure 6b,c). Consistently, RDLot‐ABE treatment significantly reduced *Fscn1* protein levels in primary mice synovial fibroblasts relative to controls (Figure 6d,e). In mice bone marrow‐derived mesenchymal stem cells (BMSCs), *Fscn1* protein levels increased progressively during osteogenic differentiation, but were markedly suppressed by RDLot‐ABE treatment (Figure 6f–i). Collectively, these results demonstrate that RDLot‐ABE enables efficient splice‐site disruption–based silencing of *Fscn1* at the protein level across multiple OA‐relevant joint cell types.

**FIGURE 7:**
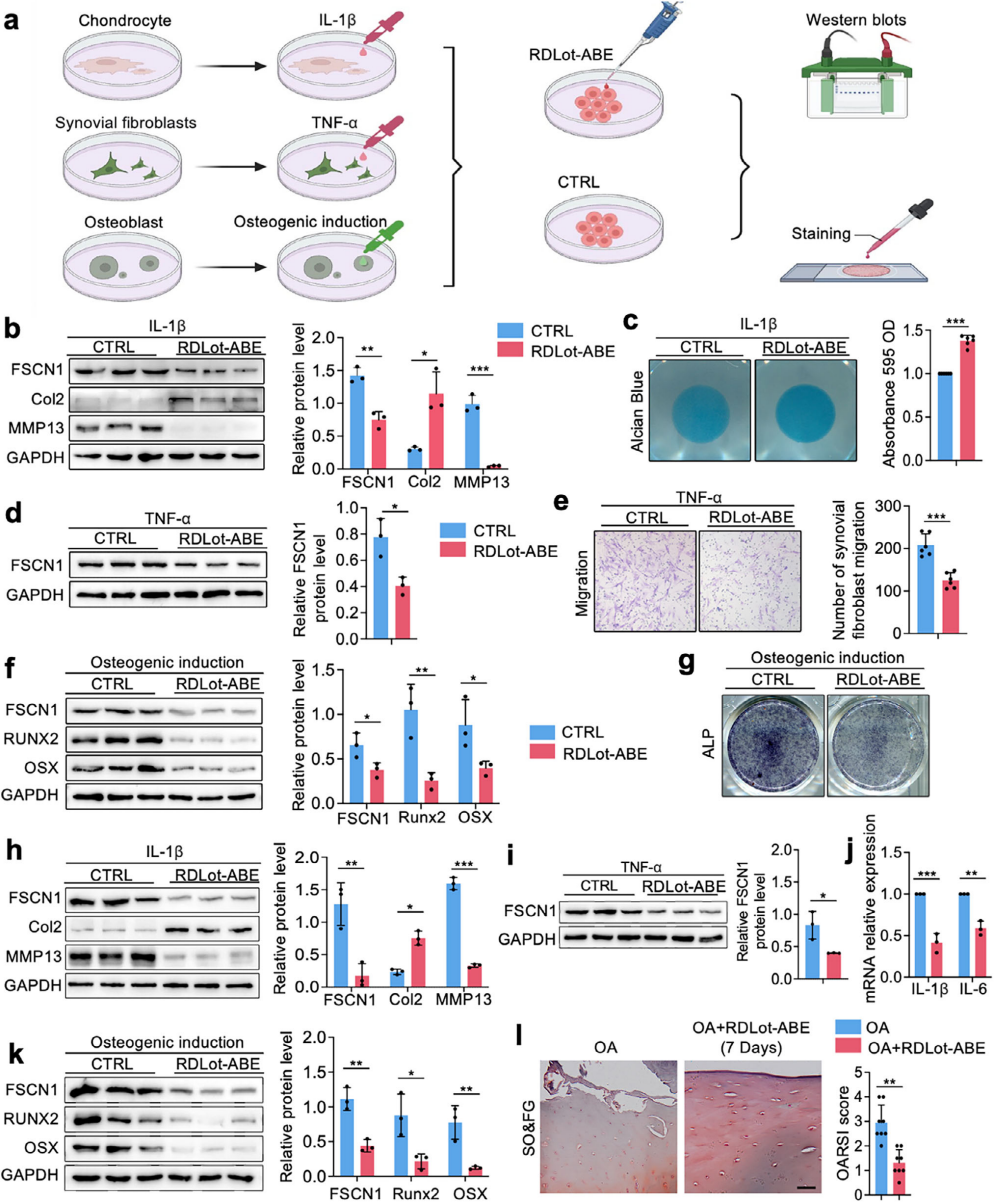
The efficacy of RDLot‐ABE lentiviral therapy in silencing *Fscn1* in inflamed chondrocytes and synovial fibroblasts, osteoblasts, and human OA cartilage explants. (a) Schematic illustration of the experimental setup: mice chondrocytes were stimulated with IL‐1β, mice synovial fibroblasts with TNF‐α, and mice BMSCs were subjected to osteogenic induction, followed by treatment with CTRL or RDLot‐ABE, and subjected to Western blot or staining. (b) Western blot and quantitative analysis of FSCN1, Col2, and MMP13 protein levels in IL‐1β‐treated mice chondrocytes with or without RDLot‐ABE treatment. (c) Alcian Blue staining and quantification in IL‐1β‐treated mice chondrocytes with or without RDLot‐ABE treatment. (d) Western blot and quantification of FSCN1 protein levels in TNF‐α‐treated mice synovial fibroblasts with or without RDLot‐ABE treatment. (e) Cell wound scratch assay and quantification of migrated mice synovial fibroblasts treated with TNF‐α, with or without RDLot‐ABE. Scale bar, 50 µm. (f,g) Western blot and quantitative analysis of FSCN1, RUNX2, and OSX protein levels, and ALP staining in osteogenic‐induced mice BMSCs treated with CTRL or RDLot‐ABE. (h) Western blot and quantitative analysis of protein levels of FSCN1, Col2, and MMP13 in human C28/I2 pretreated with IL‐1β and transduced with CTRL or RDLot‐ABE. (i,j) Western blot analysis of FSCN1 protein levels and qPCR analysis of IL‐1β and IL‐6 mRNA levels in human FLS stimulated with TNF‐α and treated with CTRL or RDLot‐ABE. (k) Western blot and quantitative analysis of FSCN1, RUNX2, and OSX protein levels in MG‐63 undergoing osteogenic induction and treated with CTRL or RDLot‐ABE. (l) Safranin O/Fast Green staining and OARSI scoring of human OA cartilage explants cultured with or without RDLot‐ABE for 7 days. Scale bar: 100 µm. Experiments were repeated three times (b,d,f–k) and six times (c,e) with similar results. *n* = 8 (l) explants per group. Results from one representative replicate are shown. Quantitative data are shown as means ± s.d. The number of biological replicates per group and *p*‐values are indicated. Statistical significance was defined as ns, not significant; *p < 0.05, **p < 0.01, ***p < 0.001.

Next, we evaluated the functional effects of RDLot‐ABE‐mediated FSCN1 suppression in multiple in vitro osteoarthritis‐relevant joint cell models (Figure 7a). In IL‐1β‐stimulated mice chondrocytes, RDLot‐ABE treatment markedly reduced *Fscn1* protein levels by approximately 27% and attenuated catabolic responses, as evidenced by an approximately 83% reduction in MMP13 protein levels. Conversely, the anabolic marker COL2A1 was restored, increasing by approximately 94% compared with control cells (Figure 7b). Consistent with these changes, Alcian blue staining revealed a marked preservation of cartilage matrix proteoglycans in RDLot‐ABE‐treated chondrocytes under IL‐1β stimulation, with staining intensity increased by approximately 48% compared with controls (Figure 7c). In TNF‐α‐stimulated mice synovial fibroblasts, RDLot‐ABE induced a reduction of >40% in *Fscn1* protein levels compared with control cells. Consistently, migration assays showed a significant suppression of synovial fibroblast migratory capacity following RDLot‐ABE treatment under TNF‐α stimulation (Figure 7d,e). Furthermore, during osteogenic induction of BMSCs, RDLot‐ABE treatment reduced FSCN1 protein levels together with the osteogenic markers RUNX2 and OSX (Figure 7f). Accordingly, alkaline phosphatase (ALP) staining revealed a marked decrease in osteogenic activity in RDLot‐ABE‐treated cells compared with controls (Figure 7g). Collectively, these findings demonstrate that RDLot‐ABE‐mediated silencing of *Fscn1* functionally ameliorates key pathological cellular phenotypes associated with osteoarthritis, including cartilage matrix degradation, synovial fibroblast migration, and aberrant osteogenic activity.

### 2.6 Potential Therapeutic Effects of RDLot‐ABE in Clinically Relevant OA Model

To bridge the gap between murine findings and clinical application, we evaluated the therapeutic efficacy of RDLot‐ABE in OA using human chondrocytes (C28/I2), human fibroblast‐like synoviocytes (FLS), human osteoblast‐like cells (MG‐63), as well as cartilage explants obtained from patients undergoing total knee arthroplasty.

In C28/I2 cells stimulated with IL‐1β, RDLot‐ABE treatment markedly reduced FSCN1 protein levels, accompanied by suppression of catabolic responses and enhancement of anabolic activity (Figure 7h). In TNF‐α–stimulated FLS cells, RDLot‐ABE treatment significantly decreased FSCN1 protein expression(Figure 7i), together with a marked reduction in the release of inflammatory mediators, including IL‐1β and IL‐6 (Figure 7j). Similarly, during osteogenic induction of MG‐63 cells, RDLot‐ABE treatment led to downregulation of FSCN1 protein levels and significantly inhibited osteogenic differentiation (Figure 7k).

Critically, we validated these findings using OA patient‐derived cartilage explants. After seven days of culture with RDLot‐ABE, histological analysis revealed mitigated proteoglycan loss, accompanied by a significant reduction in OARSI scores, indicating that cartilage matrix integrity has been maintained (Figure 7l). Together, these results demonstrate that RDLot‐ABE‐mediated *FSCN1* silencing confers disease‐modifying effects across multiple human OA‐relevant cell types and cartilage explants and provide strong preclinical support for its clinical translation.

### 2.7 Intra‐Articular Administration of RDLot‐ABE Maintains Chondrocytes Phenotype in Mouse OA Model

**FIGURE 8:**
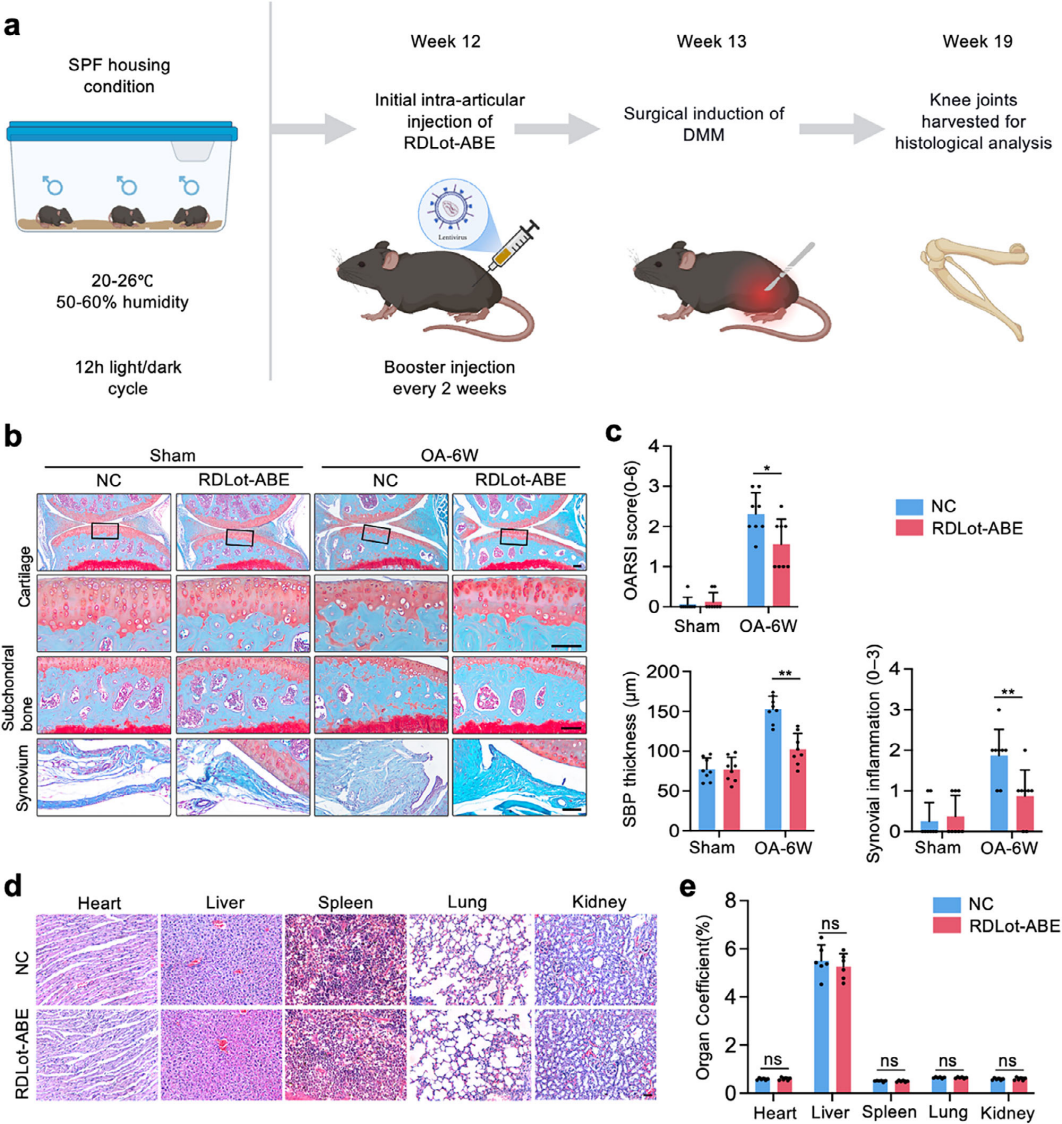
The effectiveness of RDLot‐ABE lentivirus therapy in mouse OA by silencing Fscn1. (a) Schematic of the experimental design to determine the effects of RDLot‐ABE on OA progression in 12‐week‐old C57BL/6 mice with surgically induced DMM OA. (b,c) Safranin O/Fast Green staining (b), OARSI score, subchondral bone plate (SBP) thickness, and synovial inflammation score (c) in C57BL/6 mice treated with or without RDLot‐ABE. Scale bar: 50 µm. (d,e). H&E staining (d) and organ coefficient analysis (e) of heart, liver, spleen, lung, and kidney tissues in C57BL/6 mice treated with or without RDLot‐ABE. Scale bar: 50 µm. n = 8 (b,c) or n = 6 (d,e) mice per group. Results from one representative replicate are shown. Quantitative data are shown as means ± s.d. The number of biological replicates per group and the *p*‐values are indicated. Statistical significance was defined as ns, not significant; *p < 0.05, **p < 0.01.

Encouraged by the efficacy of the RDLot‐ABE in vitro, we next evaluated the therapeutic potential of RDLot‐ABE in a murine model of OA. The model was established via destabilization of the medial meniscus (DMM) surgery in 12‐week‐old C57BL/6 mice. Intra‐articular injections of lentiviral vectors expressing RDLot‐ABE were administered starting one week prior to surgery, followed by booster injections every two weeks. Knee joint tissues were collected six weeks post‐surgery for analysis (Figure 8a). Histological staining revealed that RDLot‐ABE did not affect the normal appearance of knee cartilage in sham‐operated mice. However, in the DMM‐induced OA group, RDLot‐ABE treatment significantly mitigated cartilage degeneration, reduced subchondral bone sclerosis, and alleviated synovial inflammation compared to the control group (Figure 8b,c). Consistently, immunostaining analyses demonstrated that RDLot‐ABE treatment markedly suppressed the expression of the catabolic markers MMP3 and MMP13, while restoring the levels of the anabolic cartilage matrix components Col2a1 and aggrecan in the articular cartilage of OA mice (Figure S4a,b). These results demonstrate that RDLot‐ABE mitigates OA progression by directly targeting FSCN1, providing key preclinical evidence supporting its potential for clinical translation in gene therapy.

The in vivo safety profile of RDLot‐ABE was evaluated via comprehensive histological analysis of major organs and organ coefficient measurements in mice. Hematoxylin and eosin (H&E) staining demonstrated that the tissue structure in RDLot‐ABE‐treated mice was indistinguishable from that of controls in the heart, liver, spleen, lung, or kidney. No evidence of systemic toxicity, such as inflammatory cell infiltration, necrosis, or fibrosis, was obtained. Consistent with these histological findings, the organ coefficients for all examined tissues were comparable between groups (Figure 8d,e), which supports that RDLot‐ABE administration does not induce significant organ damage or systemic adverse effects. These findings reflect the potential of RDLot‐ABE as a safe and effective tool for clinical intervention.

## 3 Discussion

Base editing technologies hold transformative potential for correcting pathogenic point mutations, offering a precise therapeutic avenue for genetic diseases. However, the clinical translation of these powerful tools, particularly high‐activity variants like ABE8e, is currently impeded by their broad off‐target profiles, including both Cas9‐dependent genomic off‐targets and sgRNA‐independent transcriptomic deamination. To mitigate these safety risks, previous engineering efforts have predominantly concentrated on introducing mutations near the active site or the direct DNA‐binding interface. In this study, we present a synergistic strategy for engineering high‐fidelity ABE by integrating rational structural modification with PLMs‐based screening to streamline the discovery process. By strategically truncating amino acids at the non‐interactive region, we successfully engineered RDLot‐ABE variants that exhibit reduced off‐target activity while maintaining effective editing capabilities.

Consistent with previous precise or minimal off‐target variants, the optimization of off‐target effects in our RDLot‐ABE was accompanied by a trade‐off in on‐target kinetics compared to the hyperactive ABE8e. Specifically, RDLot‐ABE exhibits a refined editing window of A_4_–A_7_, which is narrower and more precise than the ABE8e(A_2_–A_10_) or the ABE9e(A_2_–A_9_), yet retains greater flexibility than the PNLM‐pcABE (A_5_–A_8_) or ABE9 (A_5_–A_6_). Notably, despite its significantly enhanced fidelity, RDLot‐ABE maintains an overall editing efficiency that shows no significant difference compared to ABE8e and ABE9e. Crucially, this safety profile was rigorously confirmed via high‐throughput sequencing assays, and the therapeutic potential was further demonstrated by the successful correction of pathogenic mutations in an OA mouse model. While this specific variant may represent just one point on the optimization spectrum, the methodology itself offers a new framework for base editor design. Importantly, this study demonstrates that distal structural elements are a viable dimension for optimization, a finding consistent with recent research conclusions [37]. This suggests that future engineering efforts need not be confined to the active site but can leverage the entire protein architecture to fine‐tune editing properties, potentially combining distal truncation with proximal mutations to balance activity and specificity more effectively.

While our current approach, combining rational design with discriminative protein models, effectively optimized the local fitness landscape of the wild‐type enzyme, it remains constrained by the inherent backbone and sequence of natural TadA. The reliance on surface geometric constraints and polar interactions means that our strategy cannot fully explore the vast, non‐continuous sequence space required to discover novel structural folds with superior intrinsic properties. Future research must therefore transcend these boundaries by integrating generative protein language models with structure‐based feature encoders to expand the search space beyond existing scaffolds. In conclusion, by validating the efficacy of AI‐assisted distal truncation, this study not only provides a specific set of safer editors for clinical translation but also establishes a transformative design framework for developing next‐generation genome editing technologies.

## 4 Methods

### 4.1 Construction of TadA‐8e Truncation Variants Library

Based on the spatial proximity to the DNA substrate and catalytic residues, three critical interaction domains were defined as “immutable”: residues 45–61, 83–91, 104–118, and 149–157. To achieve active‐site spatial constriction, we targeted the intervening loop segments connecting these secondary structures for deletion. The N‐terminus and C‐terminus were also designated as flexible regions for truncation.

A systematic deletion strategy was applied to the three intervening segments and the two termini. For each targeted segment, a sliding window approach was used to generate varied deletion lengths provided that the deletions did not encroach upon the pre‐defined conserved domains. We utilized a custom Python script to perform the combinatorial assembly of these perturbations. By coupling diverse N‐ and C‐terminal truncations with localized internal deletions, we generated a comprehensive virtual library comprising 1674 unique TadA‐8e variants.

### 4.2 Protein Language Model

The model consists of a structure encoder and a sequence encoder. In the sequence encoder section, we used the ESM‐2 650M , which is a BERT encoder developed by MetaAI to encode the protein sequence into feature vectors [18]. In the structural representation of proteins, C_α_ are defined as nodes. Sequential edges are formed between adjacent nodes, while virtual edges are introduced to represent spatial proximity between neighboring atoms(10Å). Additionally, supplementary edges are incorporated based on the k‐nearest neighbor algorithm, where k is set to 10. We denoted this graph as *G* and *R*, *E*, *T* denoted the sets of residues, edge and edge types. The method of the model follows,

(1) $$E^{\left(seq\right)}=\left\{\left(i,\;j\right)|i,j\in R,\;\left|j-i\right|<3\right\}$$

(2) $$E^{\left(radius\right)}=\left\{\left(i,\;j\right)|i,j\in R,\;\left|x_{j}-x_{i}\right|<10Å\right\}$$

(3) $$E^{\left(knn\right)}=\left\{\left(i,\;j\right)|i,j\in R,j\in knn\left(i\right)\right\}$$

(4) $$E=E^{\left(seq\right)}\cup E^{\left(radius\right)}\cup E^{\left(knn\right)}$$

where i and j denote different residues and *x_i_*, *x_y_* denote the coordinates of i and j. In the structure encoder section, we employed LoRA to enhance node representation without introducing a substantial number of additional parameters. During the model pretraining phase, we used the sequences and structures of AlphaFold DB for diffusion training. We perform diffusion on the protein sequence and structure separately, dispersing the structure and sequence into a normal distribution, and then gradually restoring the original sequence and structure. In our strategy, the objective of the fine‐tuning stage we used the ABE variants and the corresponding edited ssDNA data. We use a pre‐trained encoder to extract the features of ABE variants, fuse the ssDNA data, and then output the prediction for each position through a linear layer.

### 4.3 Low Rank Adaptation

To efficiently adapt the pre‐trained model to our downstream task, we employed Low‐Rank Adaptation, a parameter‐efficient fine‐tuning strategy. Specifically, for a pre‐trained weight matrix $W_{0}\in R^{d\times k}$ the weight update is constrained by a low‐rank decomposition *W*_0_  +  Δ*W*  = *W*_0_  + *BA*, where $B\in R^{d\times r}$ and $A\in R^{r\times d}$ are trainable matrices with rank r≪min(d,k). This approach significantly reduces the number of trainable parameters and computational memory requirements while maintaining model performance comparable to full fine‐tuning.

### 4.4 Diffusion‐Based Pre‐Training

To enhance the model's understanding of structural fitness, we performed self‐supervised pre‐training on the AlphaFold Database (AFDB) using a diffusion‐based denoising objective. During pre‐training, Gaussian noise was added to the residue coordinates, and a percentage of the sequence tokens were masked. The model was trained to reconstruct the original structure and sequence by minimizing a combined denoising loss:

$$L_{\mathrm{total}}=L_{\mathrm{MSE}}\left(Structure\right)+L_{\mathrm{CE}}\left(Sequence\right)$$

This phase allowed the model to learn a robust prior of the protein structural landscape before task‐specific fine‐tuning.

### 4.5 EC Classification

The model's functional representational capability was evaluated on the Enzyme Commission (EC) classification benchmark [28]. This dataset comprises protein structures annotated with EC numbers across 482 distinct functional categories. We followed a rigorous data splitting protocol, where the dataset was randomly partitioned into training, validation, and test sets with a ratio of 80%, 10%, and 10%, respectively. The classification performance was quantified using the F1 score (the harmonic mean of precision and recall). These metrics were chosen for their robustness in handling the inherent class imbalance across the 482 enzyme categories.

### 4.6 Protein Mutation Stability Prediction

To evaluate the model's capacity to capture the fitness implications of single‐site amino acid substitutions, we applied LoRA‐Gear to the protein mutation stability prediction task [27]. We used a three‐layer Multi‐Layer Perceptron (MLP) regression head integrated after the multimodal fusion layer. Although the primary output was a continuous score, performance was quantified using the Area Under the Receiver Operating Characteristic curve (AUROC) to remain consistent with standard benchmarks. In this context, a stability threshold was applied to the experimental values to categorize mutations as “stabilizing/neutral” or “destabilizing,” and the model's regression scores were evaluated on their ability to binary classify these variants.

### 4.7 Virtual Library Scoring

A total of 1674 structures were generated and predicted using ESMFold. Structures with a pLDDT below 80 were subsequently filtered out, leaving a total of 1036 structures. The model utilized the pre‐trained LoRA‐Gear architecture. Evolutionary features were extracted via ESM‐2, while structural constraints induced by truncations were captured using a Geometric Graph Encoder. Target DNA sequences, including the 20‐nt spacer and flanking regions, were encoded using a one‐hot encoding scheme to represent the four‐nucleotide identities as binary vectors. The latent vectors from the protein and DNA encoders were concatenated and fed into a series of fully connected layers. To adapt the pre‐trained LoRA‐Gear for the specific task of base editing, we employed Low‐Rank Adaptation (LoRA). The model was trained to output a continuous regression score representing the predicted editing efficiency for each protein‐DNA pair. To estimate the editing specificity of a single‐guide RNA, we calculated the ratio of off‐target efficiency to on‐target efficiency (off: on‐target ratio). This metric allowed for the comparison of specificity across different variants and targets, independent of the original matched target's absolute efficiency. Subsequently, the structures were further filtered via Rosetta, with the top 300 variants possessing the lowest free energy prioritized for further screening. Finally, the LoRA‐Gear uesed to identify these candidates, selecting 5 top‐ranked variants for sbusequent experimental validation.

### 4.8 Plasmid Construction

The primers and plasmid DNA sequences employed in this research are listed in the supplementary sequences of the supplementary information. The ABE8e (#138489) and lentiCRISPR v2 (#52961) plasmidswere obtained from Addgene. Polymerase chain reaction (PCR) was conducted using KOD‐Plus‐Neo DNA Polymerase (TOYOBO, Code: KOD‐401). The plasmids generated in this article, including those based on ABE8e or lentiCRISPR v2 backbones, were constructed using the ClonExpress MultiS One Step Cloning Kit (Vazyme) (Sequence S1). The construction of sgRNA expression plasmids was performed in accordance with the methodology previously described8. In brief, the oligonucleotides from Table S1 were annealed at 95 °C for 5 min, then cooled to room temperature and ligated into BbsI‐linearized vectors for sgRNA (Thermo Fisher Scientific).

### 4.9 Human Cell Culture

The HEK293T cell line (ATCC, Cat. No. CRL‐3216; RRID: CVCL_0063), human chondrocyte cell line C28/I2 (RRID: CVCL_0187), human fibroblast‐like synoviocytes (FLS; RRID: CVCL_K103), and human osteosarcoma cell line MG‐63 (ATCC, Cat. No. CRL‐1427) were purchased from Procell (Wuhan, China). HEK293T cells were maintained in Dulbecco's Modified Eagle's Medium (DMEM; Gibco) supplemented with 10% (v/v) fetal bovine serum (FBS; Gibco) and 1% penicillin–streptomycin (Gibco). C28/I2 human chondrocytes were cultured in DMEM/F12 medium (Gibco) supplemented with 10% FBS. Human fibroblast‐like synoviocytes (FLS) were cultured using the iCell Primary Fibroblast Culture System supplemented with 10% FBS (iCell). MG‐63 cells were cultured in Minimum Essential Medium (MEM; Gibco, Cat. No. 11095080) supplemented with 10% FBS. Mouse primary articular chondrocytes were isolated from the femoral condyles and tibial plateaus of 3‐day‐old C57BL/6J mice and cultured in DMEM/F12 medium (Gibco) containing 10% FBS. Mouse primary synovial fibroblasts (SFs; iCell, Cat. No. MIC‐iCell‐s004) were cultured in the iCell Primary Fibroblast Culture System supplemented with 10% FBS (iCell; Cat. No. PriMed‐iCell‐003). Mouse primary bone marrow mesenchymal stem cells (BMSCs) were isolated from the femurs and tibias of 3‐week‐old C57BL/6J mice by flushing the bone marrow cavity with sterile PBS. Cells were cultured in DMEM (Gibco; Cat. No. 12430054) supplemented with 10% FBS. For osteogenic differentiation, BMSCs were induced in osteogenic medium containing 10 mm β‐glycerophosphate, 50 µg/mL ascorbic acid, and 100 nm dexamethasone for up to 7 days. All cells were maintained under standard culture conditions at 37°C in a humidified incubator with 5% CO_2_. No bacterial or mycoplasma contamination was detected during cell culture.

### 4.10 RNA sequencing(RNA‐Seq) Experiments

For each sample, a total of 3µg RNA was used as input for library preparations. Sequencing libraries were constructed using an NEB Next Ultra RNA Library Prep Kit for Illumina(NEB) following the manufacturer's instructuions. Library quality was rigorously evaluated on an Agilent Bioanalyzer 2100 system. Subsequently, index codes were assigned to differences sequences from individual samples. The index‐coded samples were clustered on a cBot Cluster Generarion System using the TruSeq PE Cluster Kit v3‐cBot‐HS(Illumina) per the manufacturer's protocol. Finally, the resulting libraries were sequenced on an Illumina HiSeq platform, yielding 125‐bp or 150bp paired‐end reads.

### 4.11 RNA Sequence Variant Calling and Quality Control

RNA‐seq variant calling and quality control(QC) were conducted following previously established protocols [41]. Briefly, raw FASTQ reads were pre‐processed using custom Perl scripts to remove adapter sequences and trim low‐quality bases via Trimmomatic(v0.39). Simultaneously, QC metrics‐including Q20, Q30 and GC content‐were calculated to ensure data integrity. All downstream analyses were performed using the resulting high‐quality clean data. The reference genome index was constructed using HISAT2(v2.0.5), and paired‐end reads were aligned to the GRCh38 reference assembly(Ensembl) using the same software. Single‐nucleotide variants(SNVs) were identified using GATK(v4.0). To ensure high‐confidence variant calling in base editor overexpression groups, loci were filtered to exclude sites lacking robust reference genotype calls in the corresponding cotrol group.

### 4.12 Cell Transfection

For both the on‐target and off‐target base editing experiments utilizing DNA, HEK293T cells (ATCC, Catalog: CRL‐3216; RRID: CVCL_0063) were seeded into 24‐well plates and transfected at approximately 80% confluency. Subsequently, a solution comprising 3 µl of polyethyleneimine (PEI, Polysciences), 1 µg of plasmid DNA (comprising 750 ng of the ABEs expression plasmid and 250 ng of the sgRNA expression plasmid), and serum‐free medium was added to the cells. Three days following transfection, genomic DNA was extracted using the QuickExtract DNA Extraction Solution (QE09050, Epicenter), in accordance with the manufacturer's instructions and as previously described.

### 4.13 Enhanced Orthogonal R‐Loop Assay

In this study, Cas9‐independent DNA off‐target analysis was employed the enhanced modified orthogonal R‐loop assay with substituting the dSaCas9‐sgRNA plasmid with the nSaCas9‐sgRNA plasmid at each R‐loop site. For transfection, a composite of 3 µl polyethyleneimine (PEI, Polysciences), 1 µg plasmid DNA (comprising 375 ng nSaCas9‐sgRNA plasmid, 375 ng base editor plasmid, and 250 ng sgRNA plasmid) were co‐transfected into the HEK293T cells. After a three‐day period following transfection, the cells were digested using 0.25% trypsin (Gibco). Subsequently, genomic DNA was extracted using the QuickExtract DNA Extraction Solution (QE09050, Epicenter), in accordance with the experimental protocol from the manufacturer's directions.

### 4.14 In Silico‐Predicted Off‐Target Sites by Cas‐OFFinder

The selection principle of off‐target sites was performed as depicted previously. In brief, the PAM type and target genome need to be determined at the first step on the Cas‐OFFinder website, then put 20 bp target sequences of interest into the text box to initiate the searching program for potential off‐target sites with normally setting parameters up to three nucleotides mismatches and one DNA bulge.

### 4.15 High‐Throughput DNA Sequencing(HTS)and Data Analysis

On‐ and off‐target genomic regions were amplified by PCR using primers detailed in Tables S2 and S3. High‐throughput sequencing (HTS) amplification libraries were prepared by PCR using KOD‐Plus‐Neo DNA Polymerase and site‐specific primers containing an adaptor sequence (Forward 5′‐tcgtcggcagcgtcagatgtgtataagagacag‐3′; Backward 5′‐gtctcgtgggctcggagatgtgtataagagacag‐3′) at their 5′ ends. The resulting products underwent a second PCR using primers containing different barcode sequences. Subsequently, PCR products with different tags were pooled together for deep sequencing on the Illumina HiSeq platform. In the sequencing analysis, the reference sequence was carefully curated, commencing 10 base pairs anterior to the protospacer and terminating 10 base pairs posterior to the PAM sequence. The base editing or indels efficiencies were quantified using BE‐Analyzer or CRISPResso2.

### 4.16 Mouse Experiment

All animal experiments were conducted at the Third Affiliated Hospital of Southern Medical University and the Guangdong Research Institute of Orthopedics. All C57BL/6J mice were housed under specific pathogen‐free (SPF) conditions, maintained at a temperature of 20°C–26°C, humidity of 50%–60%, and a 12‐h light/dark cycle. At 12 weeks of age, mice received intra‐articular injections of RDLot‐ABE packaged in lentiviral vectors targeting FSCN1. 5 µL of viral suspension containing 5.0 × 10^6^ TU was injected into each knee joint using a microsyringe under aseptic conditions. Booster injections were administered every two weeks. Control mice received equal volumes of PBS. At 13 weeks of age, we induced OA through Destabilization of the Medial Meniscus (DMM). Briefly, after anesthetizing the mice, the right knee was disinfected. The joint capsule adjacent to the medial side of the patellar tendon was opened to expose the intercondylar area for visualization and transection of the meniscotibial ligament. The joint capsule and skin were then closed. For the sham‐operated group, the joint capsule was opened, but the meniscotibial ligament was not transected. Six weeks after DMM surgery, the mice were euthanized by rapid cervical dislocation. The knee joints were then harvested for histological analysis.

### 4.17 Histological Analysis

Soft tissues, including the mouse heart, spleen, lungs, liver, and kidneys, were fixed in 4% paraformaldehyde for 12 h. Human cartilage samples and mouse knee joints were fixed in 4% paraformaldehyde for 48 h, followed by decalcification in 10% EDTA for 30 days. All tissues were dehydrated using an automatic tissue processor (Leica ASP300S) and embedded in paraffin. Soft tissues and human cartilage were sectioned at a thickness of 4 µm. Mouse knee joints were sectioned at 4 µm thickness with 20 µm intervals according to a standard protocol. All sections were used for histological and immunohistochemical staining. Safranin O/Fast Green staining was used to evaluate the severity of OA in human and mouse articular cartilage. Cartilage degeneration was blindly scored by two independent, experienced investigators using the Osteoarthritis Research Society International (OARSI) grading system (0–6 points) [38]. To assess subchondral bone sclerosis in mice, the average distance from the subchondral bone plate to the top of the trabecular bone was measured at five evenly distributed sites [39]. Synovitis was scored as previously described (0, no inflammation; 1, mild; 2, moderate; 3, severe) [40].

### 4.18 Histological Staining

For mouse soft tissues, including heart, liver, spleen, lung, and kidney, Sections were stained with 1% hematoxylin solution (CAS No.: 517‐28‐2, Sigma‐Aldrich, St. Louis, MO, USA)for 5 min, rinsed with running tap water, differentiated with acid alcohol for several seconds, blued in alkaline solution, and then stained with 0.5% eosin Y solution (CAS No.: 17372‐87‐1, Sigma‐Aldrich, St. Louis, MO, USA) for 1 min. For mouse knee joints and human cartilage samples, Safranin O/Fast Green staining was performed. Sections were stained with a prepared 1% Fast Green solution (CAS No.: 2353‐45‐9, Sigma‐Aldrich, St. Louis, MO, USA) for 60 s, briefly rinsed with 3% acetic acid fixative solution for 3 s, and then stained with 0.5% Safranin O solution (CAS No.: 477‐73‐6, Sigma‐Aldrich) for 30 s. Sections were washed with deionized water to remove excess stain. Finally, all sections were dehydrated, cleared, and sealed with neutral gum.

### 4.19 Immunofluorescence (IF)

Representative sections prepared as described above were deparaffinized in xylene and rehydrated through graded ethanol. Sections were then immersed in TE buffer (pH 9.0) and incubated overnight at 65°C for antigen retrieval. After antigen retrieval, sections were blocked with 1% sheep serum at 37°C for 1 h and incubated overnight at 4°C with primary antibodies diluted in 1% BSA and 0.1% Triton X‐100. The sections were subsequently incubated with Alexa Fluor 488–conjugated (Life Technologies, Cat. No. A20000) or Alexa Fluor 594–conjugated (Life Technologies, Cat. No. A10438) secondary antibodies for 1 h at room temperature in the dark. Cell nuclei were counterstained with DAPI (Thermo Fisher Scientific, Cat. No. D1306). Images were acquired using a FluoView FV1000 confocal microscope (Olympus) with FV10‐ASW Viewer software (v4.2). Quantification was performed by calculating the ratio of positive cells to total cells within multiple standardized regions of interest (ROIs) using Image‐Pro Plus (v6.0) and ImageJ (v1.8). For mouse knee joints, the ratio of positive staining area or positive cell number to the total area or total cell number was analyzed along the joint surface in the femoral condyle and tibial plateau regions. For each group (at least three samples), a minimum of three random fields of view were analyzed from three randomly selected sections per sample.

### 4.20 Synovial Fibroblast Migration

A scratch assay was performed to evaluate the migratory capacity of synovial fibroblasts. Well‐conditioned mouse synovial fibroblasts was seeded in 12‐well plates and cultured until they reached approximately 100% confluence. After removal of the culture medium, a sterile 200 µL pipette tip was used to generate a cross‐shaped scratch in each well. Subsequently, medium containing 1% FBS and RDLot‐ABE lentivirus was added. After 24 h, cells were fixed with 4% paraformaldehyde and stained with ammonium oxalate crystal violet for 10 min. The stain was washed off, and five random fields of view per well were imaged and analyzed to quantify cell migration from the edges of the scratch.

### 4.21 Alcian Blue Staining of Chondrocyte Clusters

Well‐conditioned chondrocytes or C28/I2 cells were seeded into 6‐well plates and cultured until they reached approximately 95% confluence. Cells were then digested with trypsin and resuspended in 100 µL of DMEM/F12 containing 10% FBS. High‐density cell suspensions were seeded into 12‐well plates at 20 µL per drop to form tightly connected chondrocyte clusters with a diameter of approximately 0.5 cm. After attachment for 6 h at 37°C in a humidified atmosphere with 5% CO_2_, DMEM/F12 medium supplemented with 10% FBS, and the respective intervention was added. After 24 h, cells were fixed with 4% paraformaldehyde and stained with Alcian Blue for 15–20 min, followed by washing to remove excess stain. Images were acquired using a scanner, and the average staining intensity was quantified as integrated optical density (IOD) using Image‐Pro Plus software (v6.0).

### 4.22 ALP Staining

For alkaline phosphatase (ALP) staining, differentiated osteoblasts were washed with PBS and fixed with 4% PFA for 30 min at room temperature. Cells were then stained using an Alkaline Phosphatase Staining Kit (Cat. No. P0321S, Beyotime Institute of Biotechnology, China) for 3 h at room temperature in the dark. After washing to remove excess staining solution, images were acquired using a scanner.

### 4.23 Western Blot

Cells were lysed on ice using 2× SDS lysis buffer (1 m Tris‐HCl, pH 6.8, 10% SDS, glycerol, bromophenol blue, and a Roche protease inhibitor cocktail). Protein lysates were separated by 10% SDS–polyacrylamide gel electrophoresis and transferred onto 0.22 µm PVDF membranes (Sigma, Cat. No. ISEQ00010) using a wet transfer system. Membranes were blocked with 5% skim milk at room temperature for 1 h on a rocking platform and then incubated with primary antibodies at 4°C for 12–16 h. After washing, membranes were incubated with the appropriate horseradish peroxidase‐conjugated secondary antibodies for 1 h at room temperature. Protein bands were visualized using an Ultra High Sensitivity ECL Kit (MedChemExpress, Cat. No. HY‐K1005) and captured with a chemiluminescence imaging system (Tanon, Model 5200CE).

### 4.24 RNA Extraction and Quantitative Real‐Time PCR

Total RNA was extracted from human explant synovial fibroblasts treated with TNF‐α in combination with RDLot‐ABE using TRIzol reagent (9109, TaKaRa Bio Inc., Osaka, Japan), according to the manufacturer's instructions. Total RNA was reverse‐transcribed into cDNA using a cDNA synthesis kit (R333‐01, Vazyme, Nanjing, China). Primer sequences for the target genes are listed in Table S5. Quantitative real‐time PCR was performed on a LightCycler 96 system (Roche, Basel, Switzerland) using ChamQ SYBR qPCR Master Mix (Q311‐02, Vazyme), following the manufacturer's protocol. All reactions were performed in triplicate, and relative gene expression levels were calculated using the 2^−ΔΔCt method. The primer sequences used for human IL‐1β and IL‐6 were as follows: human IL‐1β, forward 5′‐ATG ATG GCT TAT TAC AGT GGC AA‐3′ and reverse 5′‐GTCGGAGATTCGTAGCTGGA‐3′; human IL‐6, forward 5′‐ACTCACCTCTTCAGAACGAATTG‐3′ and reverse 5′‐CCATCTTTGGAAGGTTCAGGTTG‐3′.

### 4.25 Human OA Cartilage Explant Culture

Human OA articular cartilage samples were obtained from 8 patients (age 50–70 years, male) with advanced OA undergoing total knee arthroplasty. All samples were sourced from The Third Affiliated Hospital of Southern Medical University, with approval from the hospital's ethics committee and consent from the patients. Cartilage tissues were trimmed into approximately 0.5 cm^3^ cubic explants under sterile conditions and rinsed thoroughly with sterile PBS. The explants were then placed into 12‐well plates containing 4 mL of DMEM/F12 medium (Gibco, Cat No. C11330500BT) supplemented with 10% fetal bovine serum (FBS; Gibco, Cat No. 10099141C). We added RDLot‐ABE lentivirus (virus 70–74, targeting FSCN1) at a final dose of 8.89 × 10^7^ transducing units (TU) per well by adding 100 µL of virus stock solution (8.89 × 10^8^ TU/mL). We also added 16 µL of Polybrene (1 mg/mL) to reach a final concentration of 4 µg/mL. The explants were cultured at 37 °C with 5% CO_2_ for 7 days. We changed the medium and added fresh virus every 2 days. At the end of culture, we fixed the explants in 4% paraformaldehyde and prepared tissue sections. These were used for histological staining to evaluate the effect of RDLot‐ABE on OA cartilage.

### 4.26 Antibody

The following antibodies were used for immunofluorescence analysis: rabbit polyclonal anti‐collagen II antibody (1:100, ab34712; Abcam), rabbit multiclonal anti‐aggrecan antibody (1:100, ab315486; Abcam), rabbit polyclonal anti‐MMP13 antibody (1:100, ab39012; Abcam), rabbit monoclonal anti‐MMP3 antibody (1:100, ab52915; Abcam), anti‐rabbit Alexa Fluor 488 (1:400, A11008; Invitrogen, USA), and anti‐rabbit Alexa Fluor 594 (1:400, A11012; Invitrogen, USA). The following antibodies were used for Western blotting: Fascin (55k‐2) mouse monoclonal antibody (1:5000, 54545; Cell Signaling Technology), rabbit monoclonal anti‐collagen II antibody (1:2000, ab188570; Abcam), rabbit polyclonal anti‐MMP13 antibody (1:3000, ab39012; Abcam), rabbit monoclonal anti‐RUNX2 antibody (1:1000, ab192256; Abcam), rabbit monoclonal anti‐Sp7/Osterix antibody (1:1000, ab209484; Abcam), GAPDH (14C10) rabbit monoclonal antibody (1:1000, 2118; Cell Signaling Technology), peroxidase‐conjugated AffiniPure goat anti‐mouse IgG (1:5000, 115‐035‐003; Jackson ImmunoResearch, USA), and peroxidase‐conjugated AffiniPure goat anti‐rabbit IgG (1:5000, 111‐035‐003; Jackson ImmunoResearch, USA).

### 4.27 Statistical Analysis

The data are expressed as the mean ± standard deviation (SD). All of the experiments were repeated at least three times. Data were analyzed and plotted using GraphPad Prism software. Student's t‐test was used for analysis of differences between two different groups. Histological scores were treated as ordinal data and analyzed using the non‐parametric Mann‐Whitney U test. Significance level are indicated as follows: *p < 0.05, *p < 0.01, and ***p < 0.001. Non‐significant differences are indicated as ‘ns’.

### 4.28 Ethical Statement

The research was conducted in accordance with all relevant ethical regulations. All procedures involving mice were meticulously designed to align with established guidelines and have received approval from the Institutional Animal Care and Use Committees (IACUCs) at the Southern Medical University, Guangzhou, China.

### 4.29 Data Avalibalility

The raw high‐throughput sequencing that support the findings of the study have been deposited to the NCBI Sequence Read Archive(SRA) under accession number PRJNA1438022. Plasmids encoding RDLoT‐ABE will be avaliable.

The code is available under the Apache 2.0 license at Github repository (http://github.com/yao-jiawei/LoRA-Gear).

## Author Contributions

X.W., K.L., and X.Z. designed and supervised the project; J.Y. designed and completed the protein language model. Z.Z. and J.R. performed the experiments and data analysis for base editing in HEK293T cells. D.C. performed the experiments for base editing in vitro and *vivo* in mice and human cartilage explants and qRT‐PCR experiments. J.Y., Z.Z., and S.W. finished data analysis for base editing in vitro and *vivo* in mice. S.W., C.Z., S.T., X.W., K.L., and X.Z. wrote and revised the manuscript. X.Z., K.L., and X.W. supervised the project and critically reviewed the manuscript.

## Funding

National Natural Science Foundation of China (No. 82350003 to X.W., and No.82522046 to X.Z.) and the CAMS Innovation Fund for Medical Sciences (No. 2025‐I2M‐QN‐008 to X.Z., and 2025‐I2M‐XHXX‐173 to X.Z.).

## Conflicts of Interest

The authors declare no conflicts of interest.

## Supporting information




**Supporting File 1**: advs74869‐sup‐0001‐SuppMat.docx.

**Supporting File 2**: advs74869‐sup‐0002‐DataFile.xlsx.

## Data Availability

The data that support the findings of this study are available in the supplementary material of this article.
