# Supplementary material for: Design of Safe and Efficient Adenine Base Editors via Protein Language Model Screening for Osteoarthritis Treatment
Source: Adv Sci (Weinh). 2026 Apr 29;13(30):e19807. doi: 10.1002/advs.202519807 (PMC13248760; doi:10.1002/advs.202519807)
Supplement: Supplementary file 1 — Supporting File 1: advs74869‐sup‐0001‐SuppMat.docx. [file ADVS-13-e19807-s001.docx]

Supporting Information

Design of Safe and Efficient Adenine Base Editors via Protein Language Model Screening for Osteoarthritis Treatment

*Jiawei Yao, Dalin Chen, Ziyi Zhang, Jingxuan Ren, Chengcheng Zhao, Shengfang Wang, Mengyu Shang， Dawei Jiang, Yinuo Li, Su’an Tang, Kai Li*^*^, *Xiaohui Zhang*^*^*, Xiaogang Wang*^*^

**Supplementary figures**


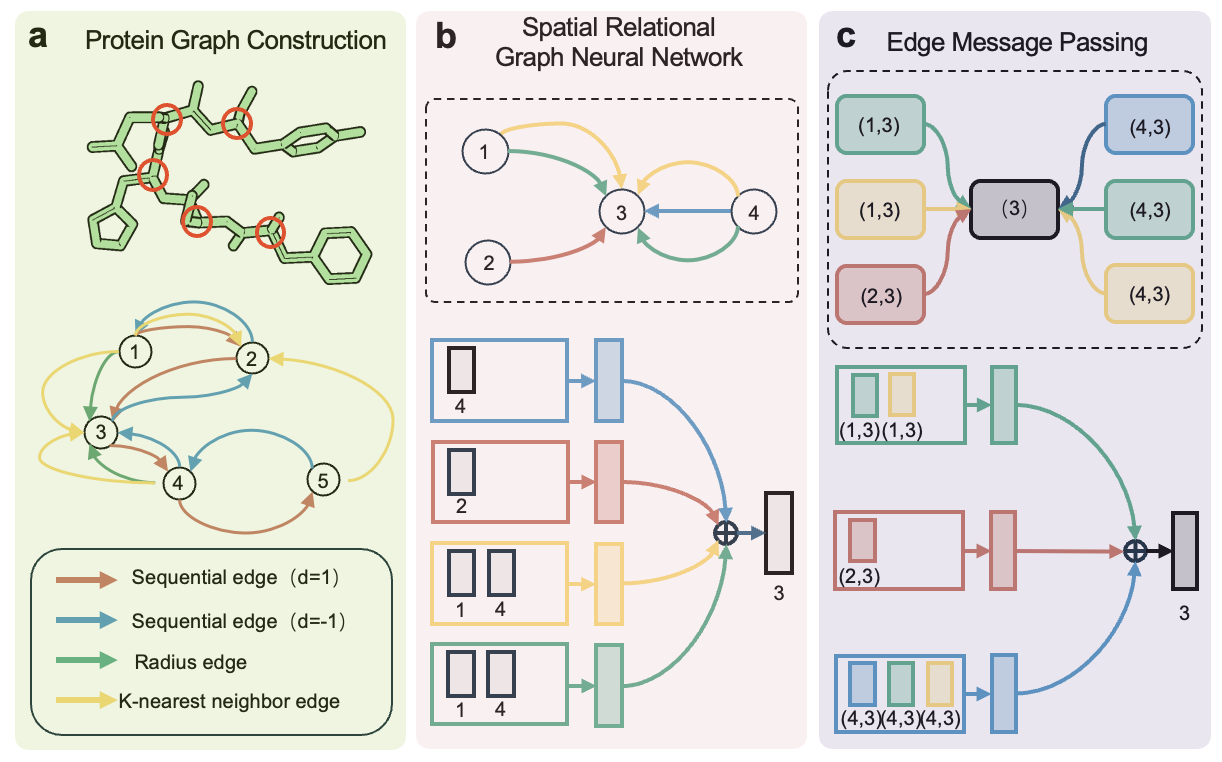


**Figure S1. The schematic of geometric graph encoder.** a. The schematic diagram of node and edge construction in graph convolutional neural networks. The protein structure is converted into a molecular graph where Cα atom of residues are represented as nodes. Edges are defined based on multiple relational criteria, including sequential connectivity (d=1, -1), spatial proximity (radius edges), and K-nearest neighbor (KNN) relationships. b. The schematic diagram of node position propagation in graph convolutional neural network. The framework utilizes a message-passing mechanism where node representations are updated by aggregating information from neighboring residues through distinct relational channels. c. The schematic diagram of edge message passing in graph convolutional neural networks. Detailed view of the message aggregation process. Messages from various edge types (e.g., (1,3), (2,3), (4,3)) are transformed and fused to update the central node (node 3). This multi-relational architecture allows the model to capture both the primary sequence context and the complex 3D spatial constraints of the protein fold.

**
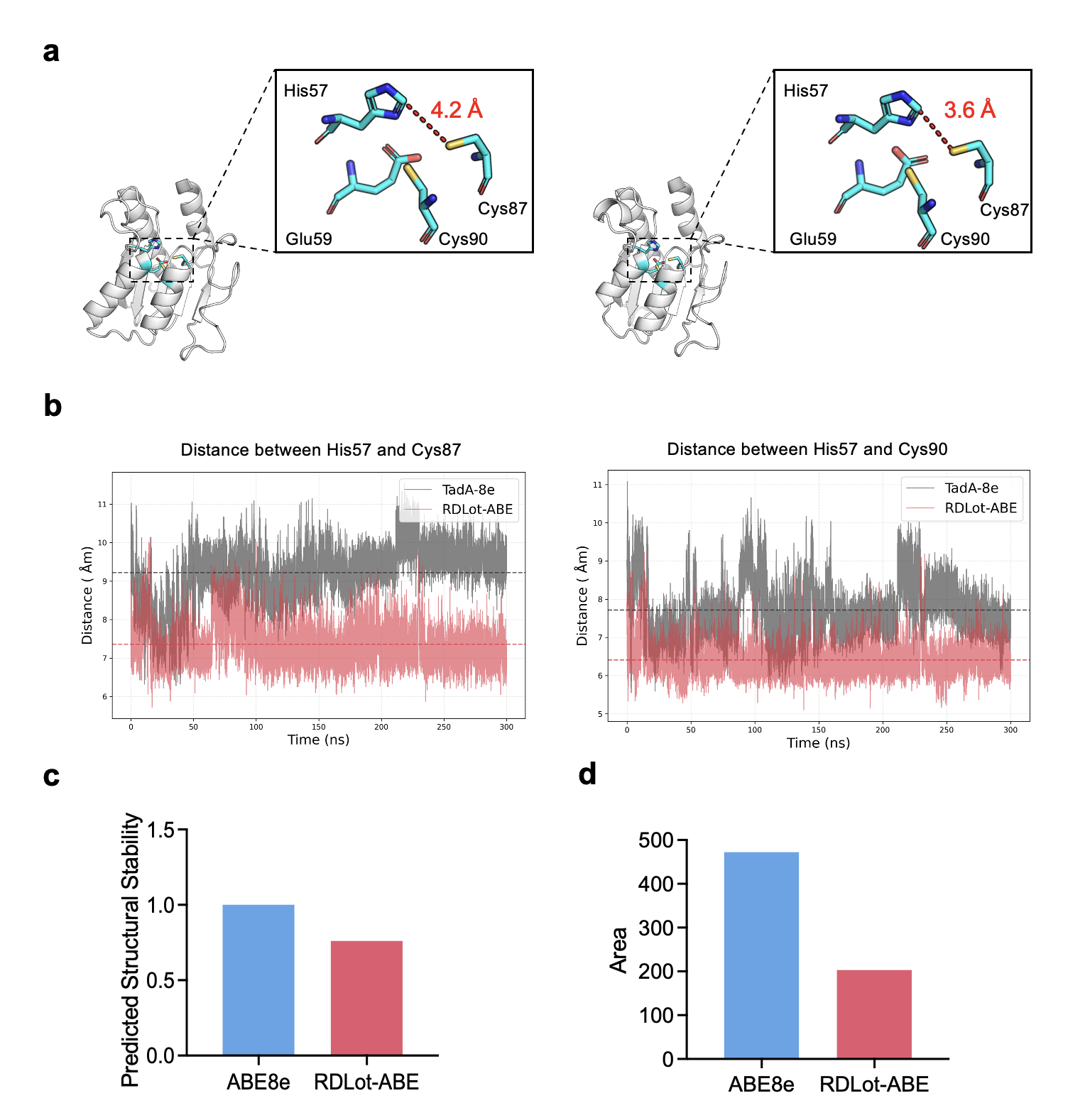
**

**Figure S2.** **Structural constriction of the RDLot-ABE catalytic pocket.** a. Structural comparison of active site pockets. The RDLot-ABE (predicted structure, right) exhibits a more constricted catalytic pocket compared to ABE8e (left). Specifically, the proximity between residues His57 and Cys87 is enhanced, with the minimum inter-atomic distance decreasing from 4.2 Å to 3.6 Å. b. Pairwise Residue-Centroid Distances Derived from MD Trajectories. MD trajectory data reveals that RDLot-ABE possesses a more constricted pocket architecture, characterized by shortened centroid distances between His57–Cys87 and His57–Cys90 relative to the ABE8e template. c. The Y-axis represents the Predicted Structural Stability of the designed variants, as quantified by thermodynamic stability scores derived from the Rosetta beta_nov16 energy function. d. To quantify the spatial constriction of the catalytic pocket, the area of the triangle formed by the Cα atoms of key residues His57, Cys87, and Cys90 was calculated. Area values are expressed in Å^2^ and were derived from the Figure S2a.

**
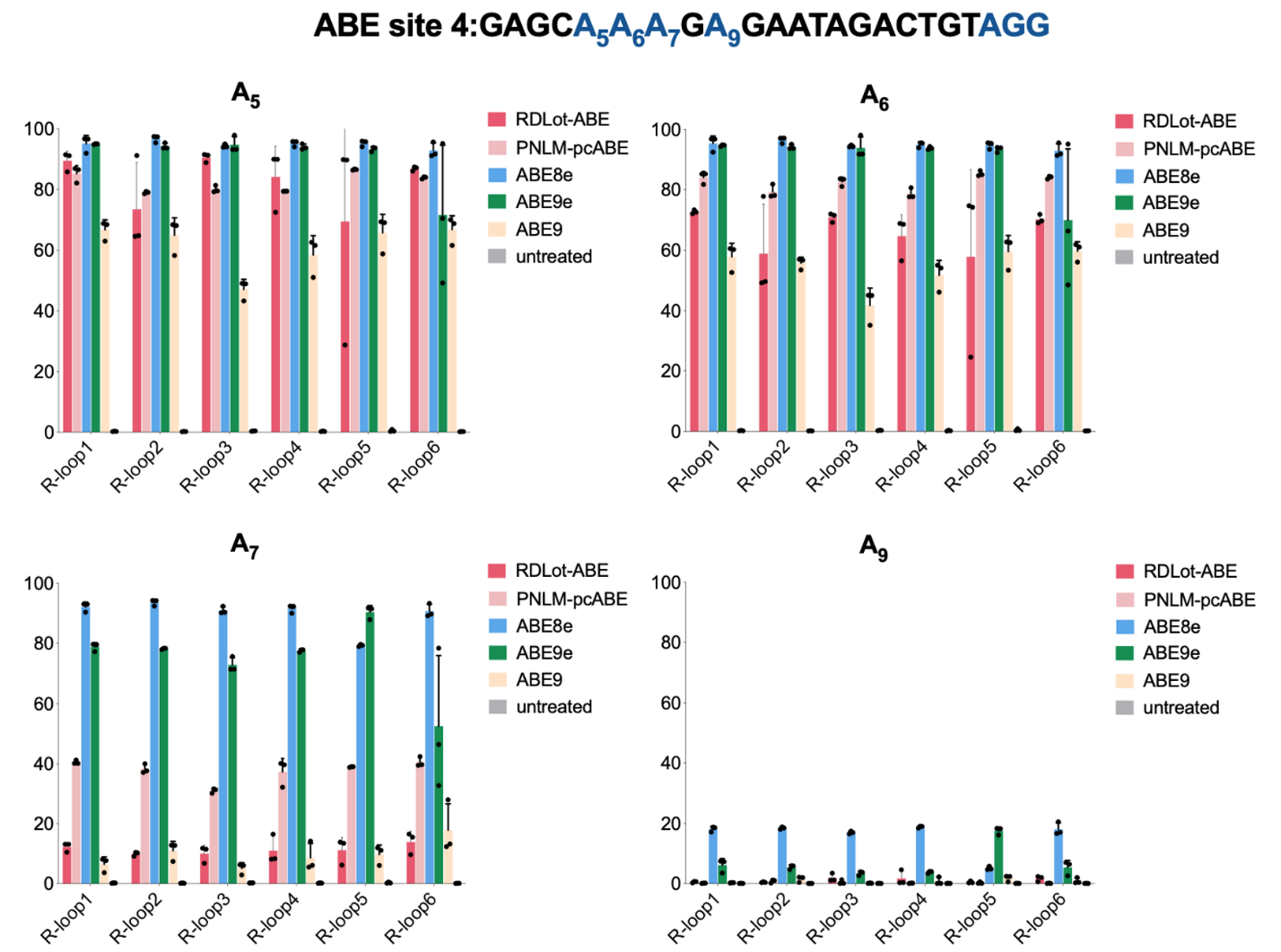
**

**Figure S3. The on-target A-to-G editing efficiency by ABEs in HEK293T cells.** The on-target A-to-G base editing efficiency of ABE8e, and RDLot-ABE at the endogenous genomic site (ABE site4) containing multiple adenosines in Fig.5d. Data are means ± s.d. (*n* = 3 independent experiments).

**
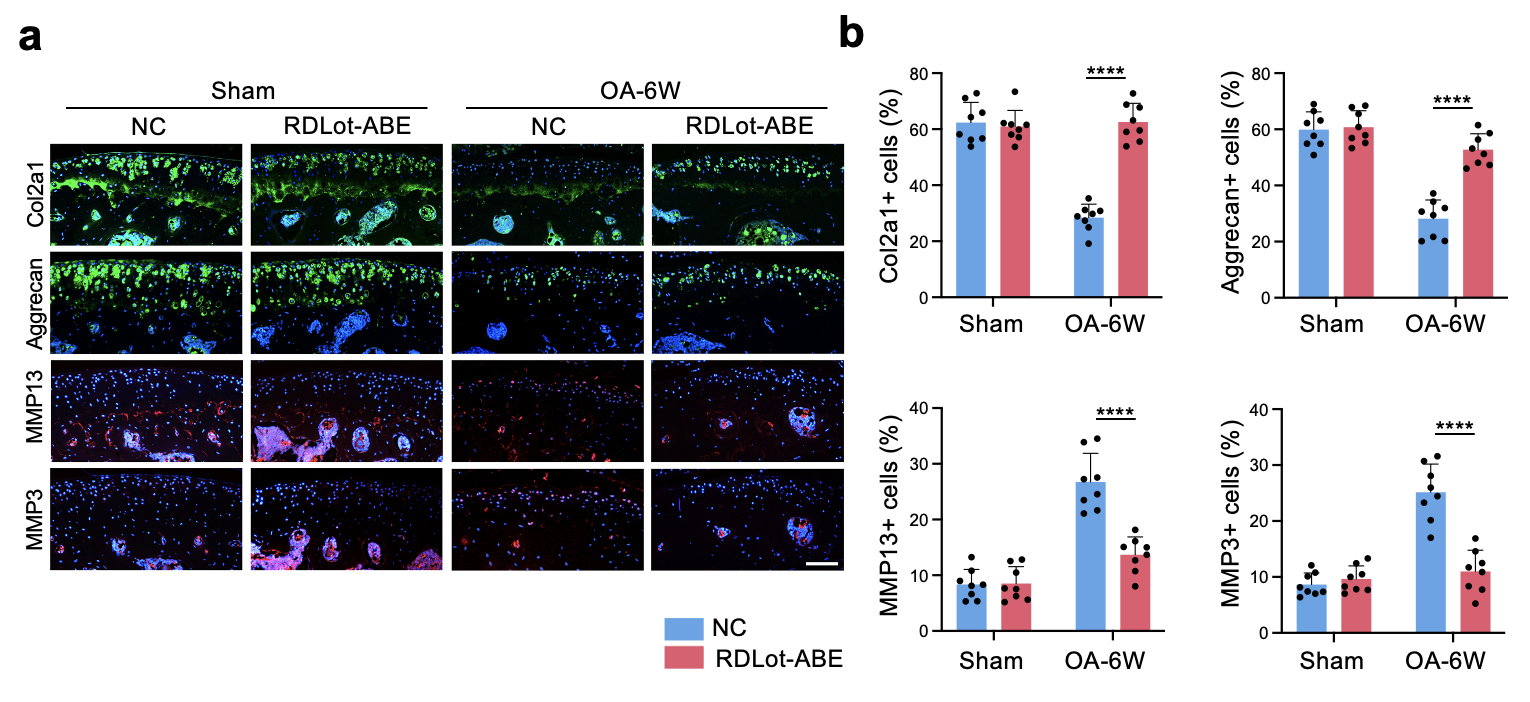
**

**Figure S4 a-b**. Immunostaining (a) and quantification (b) of Col2a1, aggrecan, MMP13, MMP3 in C57BL/6 mice treated with or without REHiFi-ABE. Scale bar: 50 μm. *n* = 8 mice per group. Results from one representative replicate are shown. Quantitative data are means ± s.d. (*n* = 8 independent experiments).

**Supplementary tables**

**Table S1.** Target protospacer sequences analyzed in this study

| **Target protospacer sequences analyzed in this study.** | | | |
| --- | --- | --- | --- |
| sgRNA | Target site sequence (5’-3’) | oligo | |
| *CCR5*-ACBE6.0-sg1p | TGACATCAATTATTATACATCGG | Oligo-up | CACCGTGACATCAATTATTATACAT |
|  |  | Oligo-dn | AAACATGTATAATAATTGATGTCAC |
| ABE site11 | GGACAGGCAGCATAGACTGTGGG | Oligo-up | CACCGGACAGGCAGCATAGACTGT |
|  |  | Oligo-dn | AAACACAGTCTATGCTGCCTGTCC |
| HEK2 | GAACACAAAGCATAGACTGCggg | Oligo-up | CACCGAGCTCACTGAACGCTGGCA |
|  |  | Oligo-dn | AAACTGCCAGCGTTCAGTGAGCTC |
| ABE site17 | gacaaagaggaagagagacgggg | Oligo-up | caccGACAAAGAGGAAGAGAGACG |
|  |  | Oligo-dn | aaacCGTCTCTCTTCCTCTTTGTC |
| *RUNX1-*ACBE6.0-sg3 | TACCCACAGTGCTTCATGAGAGG | Oligo-up | CACCGTACCCACAGTGCTTCATGAG |
|  |  | Oligo-dn | AAACCTCATGAAGCACTGTGGGTAC |
| ABE site16 | GGGAATAAATCATAGAATCCTGG | Oligo-up | CACCgggaataaatcatagaatcc |
|  |  | Oligo-dn | AAACggattctatgatttattccc |
| HEK4 | GGCACTGCGGCTGGAGGTGGggg | Oligo-up | CACCGCTTCCACATGAGCGTGGTCA |
|  |  | Oligo-dn | AAACTGACCACGCTCATGTGGAAGC |
| ABE site23 | TAAGCATAGACTCCAGGATAAGG | Oligo-up | CACCGtaagcatagactccaggata |
|  |  | Oligo-dn | AAACtatcctggagtctatgcttaC |
| *PPP1R12C* site 3 | GAGGACGTGTGTGTCTGTGTGGG | Oligo-up | CACCGAGGAGTGTGTGTCTGTGT |
|  |  | Oligo-dn | AAACACACAGACACACACGTCCTC |
| ABE site25 | AGTAAACAAAGCATAGACTGAGG | Oligo-up | CACCGagtaaacaaagcatagactg |
|  |  | Oligo-dn | AAACcagtctatgctttgtttactC |
| ABE site27 | CGGGCATCAGAATTCCCTGGAGG | Oligo-up | CACCGcgggcatcagaattccctgg |
|  |  | Oligo-dn | AAACccagggaattctgatgcccgC |
| *HBG*-sg1 | CTTGTCAAGGCTATTGGTCAAGG | Oligo-up | CACCGCTTGTCAAGGCTATTGGTCA |
|  |  | Oligo-dn | AAACTGACCAATAGCCTTGACAAGC |
| *PD1*-sg4 | CTTCCACATGAGCGTGGTCAGGG | Oligo-up | CACCGCTTCCACATGAGCGTGGTCA |
|  |  | Oligo-dn | AAACTGACCACGCTCATGTGGAAGC |
| *VEGFA* site4 | GAGCTCACTGAACGCTGGCATGG | Oligo-up | CACCGAGCTCACTGAACGCTGGCA |
|  |  | Oligo-dn | AAACTGCCAGCGTTCAGTGAGCTC |
| Notes: The italics denote gene names. | | | |

**Table S2.** PCR primers used for HTS in this study

| **PCR primers used for HTS in this study.** | | |
| --- | --- | --- |
| sgRNA | primers | primers sequence (5’-3’) |
| *CCR5*-ACBE6.0-sg1p | *CCR5*-ACBE6.0-sg1p-Hitom-F | ggagtgagtacggtgtgcAGTTTGCATTCATGGAGGGCAACTAA |
|  | *CCR5*-ACBE6.0-sg1p-Hitom-R | gagttggatgctggatggGGAGGCGGGCTGCGATTT |
| ABE site11 | ABE site11-Hitom-F | ggagtgagtacggtgtgcACTACTGCATCTTATTGCCTGC |
|  | ABE site11-Hitom-R | gagttggatgctggatggCTCCATTTTGTCTAGCTCCCAA |
| HEK2 | HEK2-Hitom-F | ggagtgagtacggtgtgcataacggaatgaatggattccttgga |
|  | HEK2-Hitom-R | gagttggatgctggatggAAACTGTGCGTATGACATCATCAGA |
| ABE site17 | ABE site17-Hitom-F | ggagtgagtacggtgtgcctcaagcctgattccaaggagatt |
|  | ABE site17-Hitom-R | gagttggatgctggatggCTCTGGTGGAGATCTTCGACTCA |
| *RUNX1-*ACBE6.0-sg3 | *RUNX1-*ACBE6.0-sg3-Hitom-F | ggagtgagtacggtgtgcAAAAGAAATCATTGAGTCCCCCGCC |
|  | *RUNX1-*ACBE6.0-sg3-Hitom-F | gagttggatgctggatggACAAGCTGCCATTTCATTACAGGCAAA |
| ABE site16 | ABE site16*-*Hitom-F | ggagtgagtacggtgtgcTCCACCTGGAATGAGTTTTCGGTTAC |
|  | ABE site16-Hitom-R | gagttggatgctggatggAATCCAGCAACACGCGGGGAG |
| HEK4 | HEK4-Hitom-F | ggagtgagtacggtgtgcAAGATGGCTGACAAAGGCCGGG |
|  | HEK4-Hitom-R | gagttggatgctggatggGAGGCGGGGGCTCAGAGAGG |
| ABE site23 | ABE site23-Hitom-F | ggagtgagtacggtgtgcacccccaaggagttccaccgcctt |
|  | ABE site23-Hitom-R | gagttggatgctggatggctcagcctctttcttcgccctctc |
| *PPP1R12C* site 3 | *PPP1R12C* site 3-Hitom-F | ggagtgagtacggtgtgccctttcaaaagaggaagctgtct |
|  | *PPP1R12C* site 3-Hitom-R | gagttggatgctggatggtggtgcgcaactgtatgaggggt |
| ABE site25 | ABE site25-Hitom-F | ggagtgagtacggtgtgcgcgtgtgaagttagctgctgccgt |
|  | ABE site25-Hitom-R | gagttggatgctggatggtaaccagctatttcatagctgtt |
| ABE site27 | ABE site27-Hitom-F | ggagtgagtacggtgtgcAATCTCAGCGCTTTCGTCCACCAC |
|  | ABE site27-Hitom-R | gagttggatgctggatggACTCCCTCCTTGGTCCAGGTTCTC |
| *HBG*-sg1 | *HBG*-sg1-Hitom-F | ggagtgagtacggtgtgcTATCCTCTTGGGGGCCCCTTCC |
|  | *HBG*-sg1-Hitom-R | gagttggatgctggatggTATTCTTCATCCCTAGCCAGCCGCC |
| *PD1*-sg4 | *PD1*-sg4-Hitom-F | ggagtgagtacggtgtgctggtaccgcatgagccccagcaa |
|  | *PD1*-sg4-Hitom-R | gagttggatgctggatggtttgatctgcgccttgggggcca |
| *VEGFA* site4 | *VEGFA* site4-Hitom-F | ggagtgagtacggtgtgcccattccctctttagccagagccgg |
|  | *VEGFA* site4-Hitom-R | gagttggatgctggatggtattggaatcctggagtgacccct |
| Notes: The italics denote gene names. | | |

**Table S3.** Off-target sites and PCR primers used for ABEs characterization

| **Off-target sites and PCR primers used for ABEs characterization.** | | | |
| --- | --- | --- | --- |
| No. | Target (5’-3’) | PCR primers for HTS | Identification method |
| *HEK* site2-Selict-seq-OT1 | AAGGAGAGGCCATAGACTGAGGG | F: ggagtgagtacggtgtgcgcaaaatgggcttggcctagga | Selict-seq |
|  |  | R: gagttggatgctggatggGCTCTCTAATCCTGCGGTTTC |  |
| *HEK* site2-Selict-seq-OT2 | CATCAGAGACTATAGACTGCAGG | F: ggagtgagtacggtgtgcaggcttaggaaggccacttcct | Selict-seq |
|  |  | R: gagttggatgctggatggcgtgaatatgtgtgggtgtgttcct |  |
| *HEK* site2-Selict-seq-OT3 | CCAGAGAGAAAATAGACTGCAGG | F: ggagtgagtacggtgtgcgctcttctcaccttttgagatgcc | Selict-seq |
|  |  | R: gagttggatgctggatgggaaagaatccgtgaactgaaacac |  |
| *HEK* site2-Selict-seq-OT4 | CTCCTAGAGGCATACACTGCGGG | F: ggagtgagtacggtgtgcgtcattcctggtcttcgtgtctg | Selict-seq |
|  |  | R: gagttggatgctggatggCTGGTCATCTCCCAATCAGGCTGG |  |
| *HEK* site2-Selict-seq-OT5 | ATATGTTGGGCATAGACTGCAGG | F: ggagtgagtacggtgtgcgggttagattattgtagtaacctt | Selict-seq |
|  |  | R: gagttggatgctggatggCTGCGCTCAACTGGAAAATCCTTTA |  |
| *HEK* site2-Selict-seq-OT6 | GAAGACACGCCCTAGACTGCAGG | F: ggagtgagtacggtgtgcCCGTTTGTAATCTTGCCATATTCTT | Selict-seq |
|  |  | R: gagttggatgctggatgggccccatgcccaggttcgcccc |  |
| *HEK* site2-Selict-seq-OT9 | TCTTAGGTGATATAGACTGCGGG | F: ggagtgagtacggtgtgcCGAAGGAGATGTCTTCAAAGGAACT | Selict-seq |
|  |  | R: gagttggatgctggatggATCTAGATAATGCAAAGCCAGGTC |  |
| *HEK* site2-GUIDE-seq-OT2 | aaacataaagcatagactgcaaa | F: gagttggatgctggatggccacaaagcagtgtagctcagggaa | GUIDE-seq |
|  |  | R: ggagtgagtacggtgtgccgagtgttattcagcctgcttggga |  |
| *HEK* site2-ChIP-seq-OT1 | ccggcagtctatgctcaccctga | F: ggagtgagtacggtgtgcccagagggagataggacaggtgagg | ChIP-seq |
|  |  | R: gagttggatgctggatggcatgcgctctgaacacaagcctttc |  |
| *HEK* site2-ChIP-seq-OT2 | gaatcgggaggggagactgcggg | F: ggagtgagtacggtgtgcgcgacctgtatccaaaagcctcaga | ChIP-seq |
|  |  | R: gagttggatgctggatggagtcaaactggaagaagagacgggc |  |
| *HEK* site2-ChIP-seq-OT3 | cctgcagtctatgcaacacttca | F: ggagtgagtacggtgtgcacccctcatgcaaatcctaacctgg | ChIP-seq |
|  |  | R: gagttggatgctggatggctccccatctctcactactgcaaaca |  |
| *HEK* site2-ChIP-seq-OT4 | ggagagagagcatagactgctgg | F: ggagtgagtacggtgtgcggaacaaacttccttggtgtgcagc | ChIP-seq |
|  |  | R: gagttggatgctggatggtgcccgaaatctaggggaagataggt |  |
| *HEK* site2-ChIP-seq-OT5 | ccagcagtctatgttttgtttgg | F: ggagtgagtacggtgtgctcattctggccctgaactgcttctg | ChIP-seq |
|  |  | R: gagttggatgctggatgggtgtggtgggcaactgttgttttgt |  |
| *PD-1*-sg4-OT1 | GTTCGAGATGAGCGTGGTCAAGG | F: ggagtgagtacggtgtgcCAAGGTTTAAATGGCAGGGCGCAG | Cas-OFFinder |
|  |  | R: gagttggatgctggatggGTGTGTGCCACCAAACCCAGCTAAT |  |
| *PD-1*-sg4-OT2 | cttccacatgagccagtgtcaagg | F: ggagtgagtacggtgtgcgggaatcgagttgttgcccaagttc | Cas-OFFinder |
|  |  | R: gagttggatgctggatgggtgcacatgtgtctgtgcatgatgt |  |
| *PD-1*-sg4-OT3 | ctgtccacatcagagtggtcatgg | F: ggagtgagtacggtgtgcgctatgagtgggcaacagcagaagg | Cas-OFFinder |
|  |  | R: gagttggatgctggatggcgtgaatatgtgtgggtgtgttcct |  |
| *PD-1*-sg4-OT4 | cttcctcatcagcgaggtcaagg | F: ggagtgagtacggtgtgcgcagggtctgtgacaaagagaagca | Cas-OFFinder |
|  |  | R: gagttggatgctggatgggggctggaaaggatagacggtcttg |  |
| *PD-1*-sg4-OT5 | ctcccacatgggcgtggttaggg | F: ggagtgagtacggtgtgcgtcctagtgggaggcaagcagc | Cas-OFFinder |
|  |  | R: gagttggatgctggatggcccccatatctcccctcaccctatc |  |
| *PD-1*-sg4-OT6 | cttccacatgggcattgtcatgg | F: ggagtgagtacggtgtgcagctgcaggtttggacttctgaaca | Cas-OFFinder |
|  |  | R: gagttggatgctggatggtggagttgatccctttgactgctcg |  |
| *PD-1*-sg4-OT7 | cttacacaagagagtggtcaggg | F: ggagtgagtacggtgtgctggcccatactcacaggctttcttg | Cas-OFFinder |
|  |  | R: gagttggatgctggatggattctgatgatgatggacctccggc |  |
| *PD-1*-sg4-OT9 | CTTCCATATGAGACGTGGTCGGGG | F: ggagtgagtacggtgtgcTGGCATATCAAACACATTCTTTCTTTGT | Cas-OFFinder |
|  |  | R: gagttggatgctggatggAGTCATACTTCTCTCCCCACAGCAA |  |
| *PD-1*-sg4-OT10 | CTTCCACATTGAACTTGGTCAAGG | F: ggagtgagtacggtgtgctaggagtgagtaaggaggggtcagc | Cas-OFFinder |
|  |  | R:gagttggatgctggatggcagaagatccacagcacaaagcagc |  |
| *HEK site4-*Tracking-seq-OT1 | GGCACgaCGGCTGGAGGTGGggg | F: ggagtgagtacggtgtgcTGTCCAAGGTCTGAGGCTCGAATCC | Tracking-seq |
|  |  | R: gagttggatgctggatggGGGGTCTCCTGTGGCCTCCA |  |
| *HEK site4-*Tracking-seq-OT2 | GGCACTGaatCTGGAGGTGGggg | F: ggagtgagtacggtgtgctgggctgtagacaggcacccg | Tracking-seq |
|  |  | R: gagttggatgctggatggctcaccaccaccaccaggaact |  |
| *HEK site4-*Tracking-seq-OT3 | GGCACTGaGaCTGGgGGTGGggg | F: ggagtgagtacggtgtgcGTTTTACCCACATCCTCACTGTGCC | Tracking-seq |
|  |  | R: gagttggatgctggatggCCCCTCCACGGGAGATGGC |  |
| *HEK site4-*Tracking-seq-OT4 | GGCAC-GCGGCTGGAGGaGGggg | F: ggagtgagtacggtgtgc AACACAGGGGCCAGTTTGTGTCTAA | Tracking-seq |
|  |  | R: gagttggatgctggatggATAACCCACAGCTGAAAGCAGAGGC |  |
| *HEK site4-*Tracking-seq-OT7 | GGCACatgGGCTGGgGGTGGggg | F: ggagtgagtacggtgtgc TTGGCATCGTCAGGGAAGTTGGG | Tracking-seq |
|  |  | R: gagttggatgctggatggATCGGGGACGCACAGGCTATGG |  |
| *HEK site4-*Tracking-seq-OT8 | GcagCTG-GGCTGGgGGTGGgGG | F: ggagtgagtacggtgtgc CTACCCCAAGTCAGCTTCC | Tracking-seq |
|  |  | R: gagttggatgctggatgggacaaagaagaaaaagagcccaa |  |
| *HEK site4-*Tracking-seq-OT9 | GGCACcG-GGgTGGAGGTGGgGG | F: ggagtgagtacggtgtgcCAATGCAGGAGGGTGGGAACCAAG | Tracking-seq |
|  |  | R: gagttggatgctggatggCATCAGCTCAGGGAAGTGTCCACAA |  |
| *HEK site4-*GUIDE-seq-OT2 | ggctctgcggctggagggggtgg | F: ggagtgagtacggtgtgctttggcaatggaggcattgg | GUIDE-seq |
|  |  | R: gagttggatgctggatgggaagaggctgcccatgagag |  |
| *HEK site4-*GUIDE-seq-OT3 | ggcacgacggctggaggtggggg | F: GGAGTGAGTACGGTGTGCGGTCTGAGGCTCGAATCCTG | GUIDE-seq |
|  |  | R: GAGTTGGATGCTGGATGGCTGTGGCCTCCATATCCCTG |  |
| *HEK site4-*GUIDE-seq-OT4 | ggcatcacggctggaggtggagg | F: GGAGTGAGTACGGTGTGCTTTCCACCAGAACTCAGCCC | GUIDE-seq |
|  |  | R: GAGTTGGATGCTGGATGGCCTCGGTTCCTCCACAACAC |  |
| *HEK site4-*GUIDE-seq-OT5 | ggcgctgcggcgggaggtggagg | F: ggagtgagtacggtgtgccacgggaaggacaggagaag | GUIDE-seq |
|  |  | R: gagttggatgctggatgggcaggggagggataaagcag |  |
| *HEK site4-*GUIDE-seq-OT6 | ggcactgagactgggggtggggg | F: GGAGTGAGTACGGTGTGCCCACGGGAGATGGCTTATGT | GUIDE-seq |
|  |  | R: GAGTTGGATGCTGGATGGCACATCCTCACTGTGCCACT |  |
| *HEK site4-*GUIDE-seq-OT7 | agcagtgcggctagaggtggtgg | F: ggagtgagtacggtgtgcgtcagtctcggcccctca | GUIDE-seq |
|  |  | R: gagttggatgctggatgggccactgtaaagctcttggg |  |
| *HEK site4-*GUIDE-seq-OT8 | ggcactgctgctgggggtggtgg | F: GGAGTGAGTACGGTGTGCAGGGTAGAGGGACAGAGCTG | GUIDE-seq |
|  |  | R: GAGTTGGATGCTGGATGGGGACCCCACATAGTCAGTGC |  |
| *HEK site4-*GUIDE-seq-OT9 | ggcactggggctgggggaggggg | F: ggagtgagtacggtgtgcgctgtcagccctatctccatc | GUIDE-seq |
|  |  | R: gagttggatgctggatggtgggcaattaggacagggac |  |
| *HEK site4-*GUIDE-seq-OT10 | ggcactggggttggaggtggggg | F: GGAGTGAGTACGGTGTGCGCAGCGGAGGAGGTAGATTG | GUIDE-seq |
|  |  | R: GAGTTGGATGCTGGATGGCTCAGTACCTGGAGTCCCGA |  |
| *HEK site4-*CHIP-seq-OT1 | aaagcagtggctggaggtggagg | F: ggagtgagtacggtgtgcgtgcaattggaggaggagct | CHIP-seq |
|  |  | R: gagttggatgctggatggcaccagctacaggcagaaca |  |
| *HEK site4-*CHIP-seq-OT3 | gagggaagggctggaggtggagg | F: ggagtgagtacggtgtgccctacccccaacacagatgg | CHIP-seq |
|  |  | R: gagttggatgctggatggccacacaactcaggtcctcc |  |
| Sa-site1 | gagtccgagcagaagaagaaggg | F: ggagtgagtacggtgtgctcatcctgcagtctcctgcttctct | R-loop |
|  |  | R: gagttggatgctggatggtctggttattttagggggatgctaggt |  |
| Sa-site2 | atttacagcctggcctttgggg | F: ggagtgagtacggtgtgcctctggtggtggccccttcct | R-loop |
|  |  | R: gagttggatgctggatggggtgcggtgggagatctggtttc |  |
| Sa-site3 | gtgtcaggtaatgtgctaaaca | F: ggagtgagtacggtgtgctggtttcttatctccttaagtgttcagc | R-loop |
|  |  | R: gagttggatgctggatggcccttcataatactacagcttaaaatgg |  |
| Sa-site4 | ggtggaggagggtgcatggggt | F: ggagtgagtacggtgtgcgagtgattgcttccaggggcctca | R-loop |
|  |  | R: gagttggatgctggatggTGTGAGCCCATCAGGTATTCAA |  |
| Sa-site5 | tttctgcttctccagccctggc | F: ggagtgagtacggtgtgcctgcgacgccctctggaggaa | R-loop |
|  |  | R: ggagtgagtacggtgtgcctgcgacgccctctggaggaa |  |
| Sa-site6 | cggatgttccaatcagtacgca | F: ggagtgagtacggtgtgcgcccagagtcaaggaacacggataa | R-loop |
|  |  | R: gagttggatgctggatgggatccaggtgctgcagaagggattc |  |
| Notes: The italics denote gene names. | | | |

**Table S4.** qPCR primers used for quantifying IL-2, IL-6 and GAPDH

| No. | qPCR primers |
| --- | --- |
| *IL-1β* | F: ATGATGGCTTATTACAGTGGCAA |
|  | R: GTCGGAGATTCGTAGCTGGA |
| *IL-6* | F: ACTCACCTCTTCAGAACGAATTG |
|  | R: CCATCTTTGGAAGGTTCAGGTTG |
| *GAPDH* | F: TGGCCTTCCGTGTTCCTAC |
|  | R: GAGTTGCTGTTGAAGTCGCA |

**Supplementary Sequences**

**Supplementary Sequences 1. DNA sequences are used is this study for cell transfection. Within base editor sequences, the bp-NLS sequence is in grey; the TadA sequence is in blue; the linker sequence is in yellow; and the Cas9 nickase sequence is in green.**

**ABE8e**

MKRTADGSEFESPKKKRKVSEVEFSHEYWMRHALTLAKRARDEREVPVGAVLVLNNRVIGEGWNRAIGLHDPTAHAEIMALRQGGLVMQNYRLIDATLYVTFEPCVMCAGAMIHSRIGRVVFGVRNSKRGAAGSLMNVLNYPGMNHRVEITEGILADECAALLCDFYRMPRQVFNAQKKAQSSINSGGSSGGSSGSETPGTSESATPESSGGSSGGSDKKYSIGLAIGTNSVGWAVITDEYKVPSKKFKVLGNTDRHSIKKNLIGALLFDSGETAEATRLKRTARRRYTRRKNRICYLQEIFSNEMAKVDDSFFHRLEESFLVEEDKKHERHPIFGNIVDEVAYHEKYPTIYHLRKKLVDSTDKADLRLIYLALAHMIKFRGHFLIEGDLNPDNSDVDKLFIQLVQTYNQLFEENPINASGVDAKAILSARLSKSRRLENLIAQLPGEKKNGLFGNLIALSLGLTPNFKSNFDLAEDAKLQLSKDTYDDDLDNLLAQIGDQYADLFLAAKNLSDAILLSDILRVNTEITKAPLSASMIKRYDEHHQDLTLLKALVRQQLPEKYKEIFFDQSKNGYAGYIDGGASQEEFYKFIKPILEKMDGTEELLVKLNREDLLRKQRTFDNGSIPHQIHLGELHAILRRQEDFYPFLKDNREKIEKILTFRIPYYVGPLARGNSRFAWMTRKSEETITPWNFEEVVDKGASAQSFIERMTNFDKNLPNEKVLPKHSLLYEYFTVYNELTKVKYVTEGMRKPAFLSGEQKKAIVDLLFKTNRKVTVKQLKEDYFKKIECFDSVEISGVEDRFNASLGTYHDLLKIIKDKDFLDNEENEDILEDIVLTLTLFEDREMIEERLKTYAHLFDDKVMKQLKRRRYTGWGRLSRKLINGIRDKQSGKTILDFLKSDGFANRNFMQLIHDDSLTFKEDIQKAQVSGQGDSLHEHIANLAGSPAIKKGILQTVKVVDELVKVMGRHKPENIVIEMARENQTTQKGQKNSRERMKRIEEGIKELGSQILKEHPVENTQLQNEKLYLYYLQNGRDMYVDQELDINRLSDYDVDHIVPQSFLKDDSIDNKVLTRSDKNRGKSDNVPSEEVVKKMKNYWRQLLNAKLITQRKFDNLTKAERGGLSELDKAGFIKRQLVETRQITKHVAQILDSRMNTKYDENDKLIREVKVITLKSKLVSDFRKDFQFYKVREINNYHHAHDAYLNAVVGTALIKKYPKLESEFVYGDYKVYDVRKMIAKSEQEIGKATAKYFFYSNIMNFFKTEITLANGEIRKRPLIETNGETGEIVWDKGRDFATVRKVLSMPQVNIVKKTEVQTGGFSKESILPKRNSDKLIARKKDWDPKKYGGFDSPTVAYSVLVVAKVEKGKSKKLKSVKELLGITIMERSSFEKNPIDFLEAKGYKEVKKDLIIKLPKYSLFELENGRKRMLASAGELQKGNELALPSKYVNFLYLASHYEKLKGSPEDNEQKQLFVEQHKHYLDEIIEQISEFSKRVILADANLDKVLSAYNKHRDKPIREQAENIIHLFTLTNLGAPAAFKYFDTTIDRKRYTSTKEVLDATLIHQSITGLYETRIDLSQLGGDSGGSKRTADGSEFEPKKKRKV

**RDLot-ABE**

MKRTADGSEFESPKKKRKVSEVEFSHEYWMRHALTLAKRARDEREVPVGAVLVLNNRVIGEGWNRAIGLHDPTAHAEIMALRQGGLVLIDATLYVTFEPCVMCAGAMIHSRIGRVVFGVRNSKRGAAGSLMNVLNYPGMNHRVEITEGILADECAALLCDFYRMPRQVFNAQKKAQSSIN

SGGSSGGSSGSETPGTSESATPESSGGSSGGS___Cas9n___SGGSKRTADGSEFEPKKKRKV

**PNLM‐pcABE**

MKRTADGSEFESPKKKRKVEYWMRHALTLAKRARDEREVPVGAVLVLNNRVIGEGWNRAIGLHDPTAHAEIMALRQGGLVMQNYRLIDATLYVTFEPCVMCAGAMIHSRIGRVVFGVRNSKRGAAGSLMNVLNYPGMNHRVEITEGILADECAALLCRQVFNAQKKAQSSIN

SGGSSGGSSGSETPGTSESATPESSGGSSGGS___Cas9n___SGGSKRTADGSEFEPKKKRKV

**ABE9e**

MKRTADGSEFESPKKKRKVSEVEFSHEYWMRHALTLAKRARDEREVPVGAVLVLNNRVIGEGWNRAIGLHDPTAHAEIMALRQGGLVMQNYRLIDATLYVTFEPCVMCAGAMIHSRIGRVVFGVRNSKTGAAGSLMNVLNYPGMKHRVEITEGILADECAALLCDFYRMPRRVFNAQKKAQSSIN

SGGSSGGSSGSETPGTSESATPESSGGSSGGS___Cas9n___SGGSKRTADGSEFEPKKKRKV

**ABE9**

MKRTADGSEFESPKKKRKVSEVEFSHEYWMRHALTLAKRARDEREVPVGAVLVLNNRVIGEGWNRAIGLHDPTAHAEIMALRQGGLVMQNYRLIDATLYVTFEPCVMCAGAMIHSRIGRVVFGVRQSKRGAAGSLMNVLNYPGMNHRVEITEGILADECAALTCDFYRMPRQVFNAQKKAQSSIN

SGGSSGGSSGSETPGTSESATPESSGGSSGGS___Cas9n___SGGSKRTADGSEFEPKKKRKV

**ABE-Δ2-8**

MKRTADGSEFESPKKKRKVEYWMRHALTLAKRARDEREVPVGAVLVLNNRVIGEGWNRAIGLHDPTAHAEIMALRQGGLVMQNYRLIDATLYVTFEPCVMCAGAMIHSRIGRVVFGVRNSKRGAAGSLMNVLNYPGMNHRVEITEGILADECAALLCDFYRMPRQVFNAQKKAQSSIN

SGGSSGGSSGSETPGTSESATPESSGGSSGGS___Cas9n___SGGSKRTADGSEFEPKKKRKV

**ABE-Δ2-21**

MKRTADGSEFESPKKKRKV

ARDEREVPVGAVLVLNNRVIGEGWNRAIGLHDPTAHAEIMALRQGGLVMQNYRLIDATLYVTFEPCVMCAGAMIHSRIGRVVFGVRNSKRGAAGSLMNVLNYPGMNHRVEITEGILADECAALLCDFYRMPRQVFNAQKKAQSSIN

SGGSSGGSSGSETPGTSESATPESSGGSSGGS___Cas9n___SGGSKRTADGSEFEPKKKRKV

**ABE-Δ2-31**

MKRTADGSEFESPKKKRKV

AVLVLNNRVIGEGWNRAIGLHDPTAHAEIMALRQGGLVMQNYRLIDATLYVTFEPCVMCAGAMIHSRIGRVVFGVRNSKRGAAGSLMNVLNYPGMNHRVEITEGILADECAALLCDFYRMPRQVFNAQKKAQSSIN

SGGSSGGSSGSETPGTSESATPESSGGSSGGS___Cas9n___SGGSKRTADGSEFEPKKKRKV

**ABE-Δ32-41**

MKRTADGSEFESPKKKRKVSEVEFSHEYWMRHALTLAKRARDEREVPVGGEGWNRAIGLHDPTAHAEIMALRQGGLVMQNYRLIDATLYVTFEPCVMCAGAMIHSRIGRVVFGVRNSKRGAAGSLMNVLNYPGMNHRVEITEGILADECAALLCDFYRMPRQVFNAQKKAQSSIN

SGGSSGGSSGSETPGTSESATPESSGGSSGGS___Cas9n___SGGSKRTADGSEFEPKKKRKV
